# Daratumumab plus bortezomib, lenalidomide and dexamethasone for transplant-ineligible or transplant-deferred newly diagnosed multiple myeloma: the randomized phase 3 CEPHEUS trial

**DOI:** 10.1038/s41591-024-03485-7

**Published:** 2025-02-05

**Authors:** Saad Z. Usmani, Thierry Facon, Vania Hungria, Nizar J. Bahlis, Christopher P. Venner, Marc Braunstein, Ludek Pour, Josep M. Martí, Supratik Basu, Yael C. Cohen, Morio Matsumoto, Kenshi Suzuki, Cyrille Hulin, Sebastian Grosicki, Wojciech Legiec, Meral Beksac, Angelo Maiolino, Hiroyuki Takamatsu, Aurore Perrot, Mehmet Turgut, Tahamtan Ahmadi, Weiping Liu, Jianping Wang, Katherine Chastain, Jessica Vermeulen, Maria Krevvata, Lorena Lopez-Masi, Jodi Carey, Melissa Rowe, Robin Carson, Sonja Zweegman

**Affiliations:** 1 Memorial Sloan Kettering Cancer Center, New York, NY USA; 2 University of Lille, CHU de Lille, Service des Maladies du Sang, Lille, France; 3 Clínica Médica São Germano, São Paulo, Brazil; 4 Arnie Charbonneau Cancer Research Institute, University of Calgary, Calgary, Alberta Canada; 5 Department of Medical Oncology, Cross Cancer Institute, University of Alberta, Edmonton, Alberta Canada; 6 BC Cancer—Vancouver Centre, University of British Columbia, Vancouver, British Columbia Canada; 7 Perlmutter Cancer Center, NYU Langone Health, New York, NY USA; 8 University Hospital Brno, Brno, Czech Republic; 9 Hospital Universitario Mútua de Terrassa, Terrassa, Spain; 10 Royal Wolverhampton NHS Trust and University of Wolverhampton, CRN West Midlands, National Institute for Health and Care Research, Wolverhampton, UK; 11 Department of Hematology, Tel Aviv Sourasky (Ichilov) Medical Center, Tel Aviv, Israel; 12 Faculty of Medical and Health Sciences, Tel Aviv University, Tel Aviv, Israel; 13 Department of Hematology, National Hospital Organization Shibukawa Medical Center, Gunma, Japan; 14 Department of Hematology, Japanese Red Cross Medical Center, Tokyo, Japan; 15 Department of Hematology, Hôpital Haut Lévêque, University Hospital, Pessac, France; 16 Department of Hematology and Cancer Prevention, School of Public Health, Medical University of Silesia, Katowice, Poland; 17 Department of Hematology and Bone Marrow Transplantation, St. John of Dukla Oncology Center of Lublin Land, Lublin, Poland; 18 Istinye University, Ankara Liv Hospital, Ankara, Turkey; 19 Instituto Americas de Ensino, Pesquisa e Inovação, Rio de Janeiro, Brazil; 20 Universidade Federal do Rio de Janeiro, Rio de Janeiro, Brazil; 21 Department of Hematology, Kanazawa University Hospital, Kanazawa University, Kanazawa, Japan; 22 CHU de Toulouse, IUCT-O, Université de Toulouse, UPS, Service d’Hématologie, Toulouse, France; 23 Department of Hematology, Ondokuz Mayıs University Faculty of Medicine, Samsun, Turkey; 24 Genmab US, Inc., Plainsboro, NJ USA; 25 Johnson & Johnson, Shanghai, China; 26 Johnson & Johnson, Spring House, PA USA; 27 Johnson & Johnson, Raritan, NJ USA; 28 Johnson & Johnson, Leiden, The Netherlands; 29 Johnson & Johnson, High Wycombe, UK; 30 Department of Hematology, Amsterdam UMC, Vrije Universiteit Amsterdam, Cancer Center Amsterdam, Amsterdam, The Netherlands

**Keywords:** Myeloma, Targeted therapies

## Abstract

Frontline daratumumab-based triplet and quadruplet standard-of-care regimens have demonstrated improved survival outcomes in newly diagnosed multiple myeloma (NDMM). For patients with transplant-ineligible NDMM, triplet therapy with either daratumumab plus lenalidomide and dexamethasone (D-Rd) or bortezomib, lenalidomide and dexamethasone (VRd) is the current standard of care. This phase 3 trial evaluated subcutaneous daratumumab plus VRd (D-VRd) in patients with transplant-ineligible NDMM or for whom transplant was not planned as the initial therapy (transplant deferred). Some 395 patients with transplant-ineligible or transplant-deferred NDMM were randomly assigned to eight cycles of D-VRd or VRd followed by D-Rd or Rd until progression. The primary endpoint was overall minimal residual disease (MRD)-negativity rate at 10^−^^5^ by next-generation sequencing. Major secondary endpoints included complete response (CR) or better (≥CR) rate, progression-free survival and sustained MRD-negativity rate at 10^−^^5^. At a median follow-up of 58.7 months, the MRD-negativity rate was 60.9% with D-VRd versus 39.4% with VRd (odds ratio, 2.37; 95% confidence interval (CI), 1.58–3.55; *P* < 0.0001). Rates of ≥CR (81.2% versus 61.6%; *P* < 0.0001) and sustained MRD negativity (≥12 months; 48.7% versus 26.3%; *P* < 0.0001) were significantly higher with D-VRd versus VRd. Risk of progression or death was 43% lower for D-VRd versus VRd (hazard ratio, 0.57; 95% CI, 0.41–0.79; *P* = 0.0005). Adverse events were consistent with the known safety profiles for daratumumab and VRd. Combining daratumumab with VRd produced deeper and more durable MRD responses versus VRd alone. The present study supports D-VRd quadruplet therapy as a new standard of care for transplant-ineligible or transplant-deferred NDMM. ClinicalTrials.gov registration: NCT03652064.

## Abstract (web summary)

In the phase 3 CEPHEUS trial, patients with transplant-ineligible or transplant-deferred newly diagnosed multiple myeloma were treated with subcutaneous daratumumab plus bortezomib, lenalidomide and dexamethasone (D-VRd), which led to a significantly deeper and more durable increase in minimal residual disease responses compared with the control arm of VRd.

## Main

Daratumumab is a human immunoglobulin (Ig)Gκ monoclonal antibody targeting CD38 with direct on-tumor^1–4^ and immunomodulatory^5–7^ mechanisms of action that has demonstrated overall survival benefit in three frontline regimens^8–10^ and was the first anti-CD38 monoclonal antibody approved in newly diagnosed multiple myeloma (NDMM)^11,12^. Frontline daratumumab-based triplet and quadruplet standard-of-care regimens have demonstrated improved survival outcomes. For transplant-ineligible patients, significant progression-free survival (PFS) benefit was observed with frontline daratumumab plus lenalidomide and dexamethasone (D-Rd) triplet therapy versus lenalidomide and dexamethasone (Rd) alone in the phase 3 MAIA study^8,13,14^, which set a new benchmark for the transplant-ineligible population, with a median overall survival of 7.5 years^14^. The phase 3 PERSEUS study (quadruplet daratumumab plus bortezomib, lenalidomide and dexamethasone (D-VRd) induction/consolidation and daratumumab–lenalidomide maintenance versus VRd induction/consolidation and lenalidomide maintenance) demonstrated that frontline treatment with daratumumab across the treatment continuum (induction/consolidation/maintenance) significantly improved PFS and increased the depth of the response versus the standard of care in the transplant-eligible setting^15^.

The phase 3 CEPHEUS study evaluated quadruplet D-VRd versus VRd alone in patients with NDMM who were transplant ineligible or for whom transplant was not planned as the initial therapy (transplant deferred). At the time the study was designed, triplet VRd therapy was a standard of care based on the SWOG S0777 trial, with CEPHEUS implementing the same VRd dosing with subcutaneous bortezomib^16,17^. Here we report results from CEPHEUS after the final PFS analysis.

## Results

### Patients and treatment

**Fig. 1:**
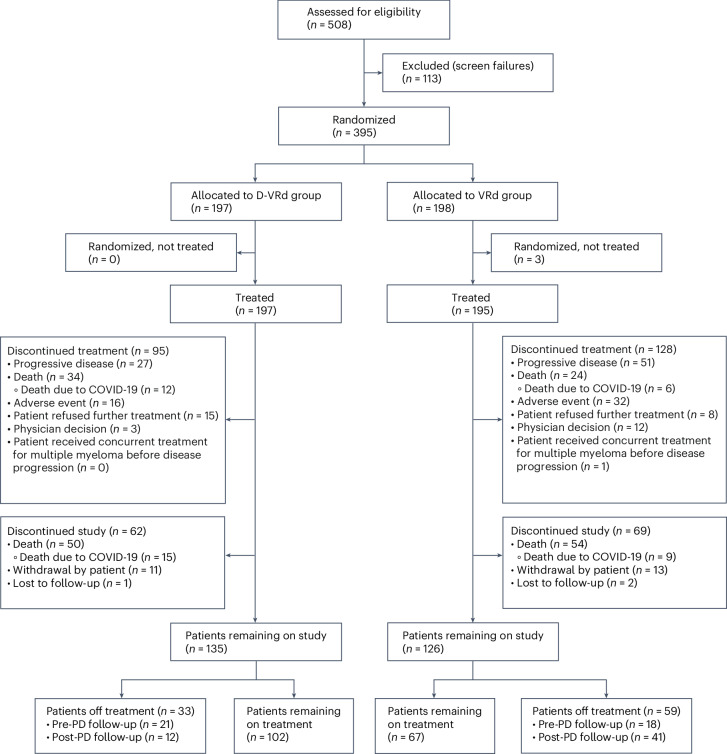
CONSORT patient flow diagram. Patient disposition at the data cutoff date of 7 May 2024.

A total of 395 patients with transplant-ineligible or transplant-deferred NDMM were enrolled between 11 December 2018 and 7 October 2019, with 197 and 198 assigned to D-VRd and VRd, respectively. Enrollment by country is summarized in Supplementary Table 1. Among randomized patients, 392 (197 for D-VRd and 195 for VRd) received ≥1 dose of the assigned treatment (Fig. 1). Demographic and baseline characteristics were generally balanced between the groups (Table 1). The median patient age was 70 years (range 31–80 years); 28.1% had International Staging System (ISS) stage III disease, and 13.2% had high cytogenetic risk (t(4;14), t(14;16) or del(17p)). The percentage of patients with an Eastern Cooperative Oncology Group (ECOG) performance status score of 2 was 11.7% for D-VRd versus 7.1% for VRd; 36.0% and 42.4%, respectively, had an ECOG performance status score of 0.

**Table 1:** Demographic and clinical characteristics in the intention-to-treat population at baseline^a^

Characteristic	D-VRd (*n* = 197)	VRd (*n* = 198)
Age		
Median (range) (years)	70 (42–79)	70 (31–80)
Distribution, no. (%)		
<65 years	36 (18.3)	35 (17.7)
65 to <70 years	52 (26.4)	53 (26.8)
≥70 years	109 (55.3)	110 (55.6)
Age/transplant eligibility, no. (%)		
<70 years and transplant ineligible	35 (17.8)	35 (17.7)
<70 years and transplant deferred	53 (26.9)	53 (26.8)
≥70 years	109 (55.3)	110 (55.6)
Male sex, no. (%)^b^	87 (44.2)	111 (56.1)
Race, no. (%)^b^		
White	162 (82.2)	156 (78.8)
Black or African American	10 (5.1)	9 (4.5)
Asian	11 (5.6)	14 (7.1)
Native Hawaiian or other Pacific Islander	0	1 (0.5)
Other	1 (0.5)	2 (1.0)
Not reported	13 (6.6)	16 (8.1)
ECOG performance status score, no. (%)^c^		
0	71 (36.0)	84 (42.4)
1	103 (52.3)	100 (50.5)
2	23 (11.7)	14 (7.1)
Frailty score, no. (%)^d^		
0 (fit)	124 (62.9)	132 (66.7)
1 (intermediate fitness)	73 (37.1)	66 (33.3)
Type of measurable disease, no. (%)		
Detected in serum only	120 (60.9)	108 (54.5)
IgG	89 (45.2)	76 (38.4)
IgA	27 (13.7)	31 (15.7)
Other^e^	4 (2.0)	1 (0.5)
Detected in serum and urine	41 (20.8)	45 (22.7)
Detected in urine only	20 (10.2)	24 (12.1)
Detected in serum free light chains only	16 (8.1)	21 (10.6)
ISS disease stage, no. (%)^f^		
I	68 (34.5)	68 (34.3)
II	73 (37.1)	75 (37.9)
III	56 (28.4)	55 (27.8)
Cytogenetic risk profile, no. (%)^g^		
Standard risk	149 (75.6)	149 (75.3)
High risk	25 (12.7)	27 (13.6)
Indeterminate^h^	23 (11.7)	22 (11.1)
Median time since diagnosis of multiple myeloma (range) (months)	1.2 (0.4–5.8)	1.3 (0.3–8.0)

^a^The intention-to-treat population was defined as all patients who underwent randomization.

^b^Sex and race were reported by the patient.

^c^ECOG performance status is scored on a scale of 0–5, with 0 indicating no symptoms and higher scores indicating increasing disability.

^d^Total additive frailty is scored on a scale of 0–5 based on age, comorbidities and cognitive and physical conditions, with 0 indicating fit, 1 intermediate fitness and ≥2 frail, per the Myeloma Geriatric Assessment score (http://www.myelomafrailtyscorecalculator.net).

^e^Includes IgD, IgM, IgE and biclonal.

^f^ISS disease stage is based on the combination of serum β_2_-microglobulin and albumin levels. Higher stages indicate more advanced disease.

^g^Cytogenetic risk was assessed by fluorescence in situ hybridization. High risk was defined as the presence of del(17p), t(4;14) and/or t(14;16).

^h^Indeterminate includes patients with missing or unevaluable samples.

At clinical cutoff (7 May 2024), 102 patients (51.8%) in the D-VRd group and 67 (34.4%) in the VRd group remained on treatment. The most common reason for treatment discontinuation was progressive disease (D-VRd, 13.7%; VRd, 26.2%).

The median duration of study treatment was 22 months longer for D-VRd compared with VRd (56.3 versus 34.3 months; Extended Data Table 1). The median number of treatment cycles was greater for D-VRd versus VRd (59 (range 1–71) versus 37 (range 1–70)). The relative dose intensities were similar between treatment arms (Extended Data Table 1).

### Efficacy

**Fig. 2:**
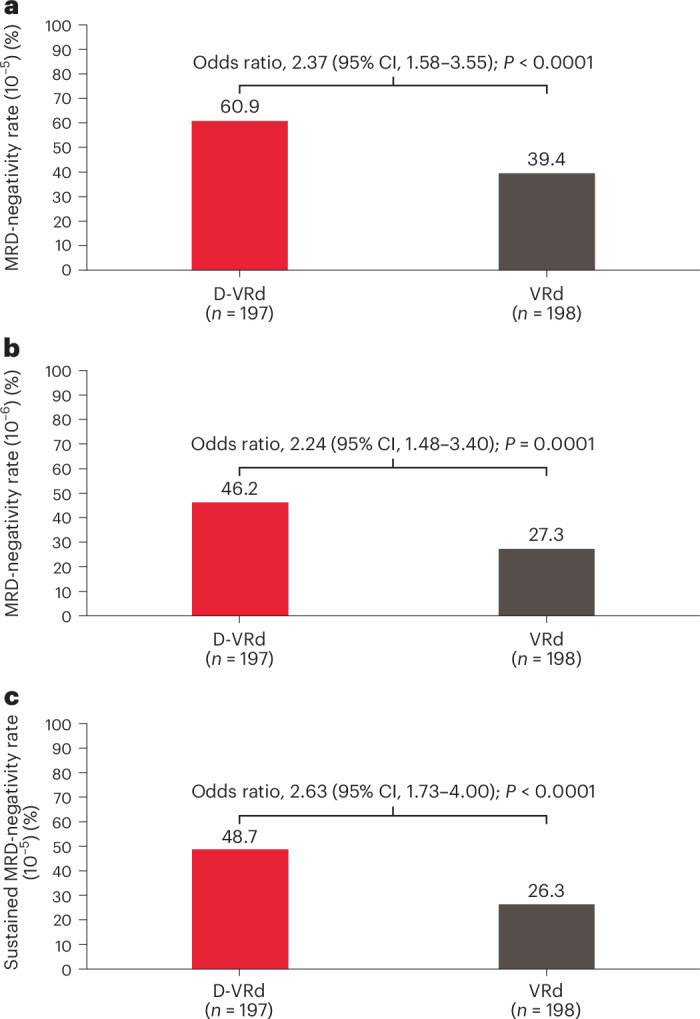
MRD-negativity rates. **a**, The primary endpoint of overall MRD-negativity rates in the intention-to-treat population. The overall MRD-negativity rate was defined as the proportion of patients who achieved ≥CR and MRD negativity (at or below a sensitivity threshold of 10^−^^5^) after randomization but before disease progression, subsequent antimyeloma therapy or both. **b**, An exploratory analysis of the MRD-negativity rate at or below a sensitivity threshold of 10^−^^6^. **c**, Sustained MRD-negativity rate in the intention-to-treat population. The sustained MRD-negativity rate was defined as the proportion of patients who achieved ≥CR and MRD-negative status (at or below a sensitivity threshold of 10^−^^5^) at two examinations a minimum of 1 year apart without MRD-positive status in between. MRD status was assessed using bone marrow samples and evaluated using a next-generation sequencing assay (clonoSEQ assay, v.2.0; Adaptive Biotechnologies) in accordance with International Myeloma Working Group guidelines for assessing MRD^25^. The Mantel–Haenszel estimate of the common odds ratio for stratified tables was used. The stratification factors were ISS disease stage (I, II or III) and age/transplant eligibility (<70 years and transplant ineligible, <70 years and transplant deferred or ≥70 years). An odds ratio >1 indicates an advantage for D-VRd. The *P* value (two sided) was calculated using a Fisher’s exact test.

**Extended Data Fig. 1:**
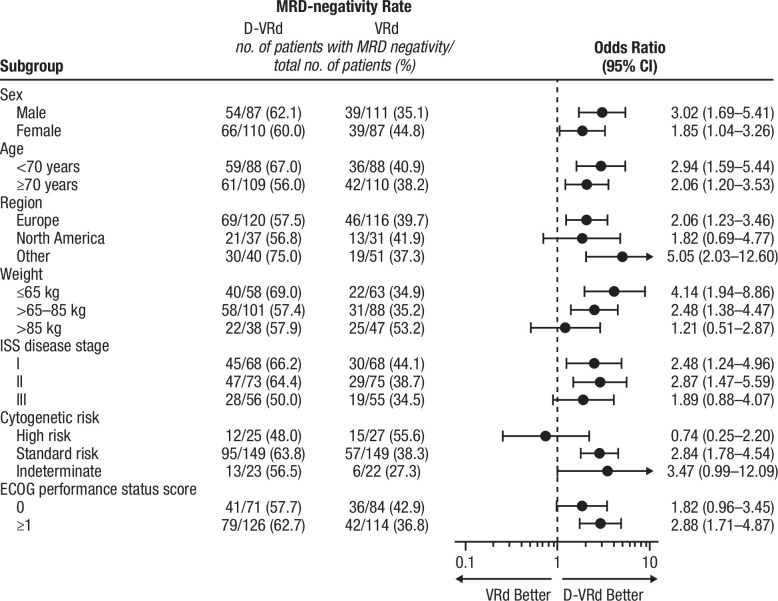
Prespecified subgroup analysis of overall MRD-negativity rate. Shown are the results of an analysis of overall MRD-negativity rate in prespecified subgroups in the intention-to-treat population. Data are presented as odds ratios and 95% CIs. Overall MRD-negativity rate was defined as the proportion of patients who achieved complete response or better and MRD negativity (at or below a sensitivity threshold of 10^–5^) after randomization but prior to disease progression, subsequent antimyeloma therapy or both. The ISS consists of three stages, with higher stages indicating more severe disease: stage I, serum β_2_-microglobulin level <3.5 mg per liter (300 nmol per liter) and albumin level ≥3.5 g per deciliter; stage II, neither stage I or III; and stage III, serum β_2_-microglobulin level ≥5.5 mg per liter (≥470 nmol per liter). Cytogenetic risk was assessed by fluorescence in situ hybridization. High risk was defined as the presence of del(17p), t(4;14) and/or t(14;16). Indeterminate includes patients with missing or unevaluable samples. CI, confidence interval; D-VRd, subcutaneous daratumumab plus bortezomib/lenalidomide/dexamethasone; ECOG, Eastern Cooperative Oncology Group; ISS, International Staging System; MRD, minimal residual disease; VRd, bortezomib/lenalidomide/dexamethasone.

**Extended Data Fig. 2:**
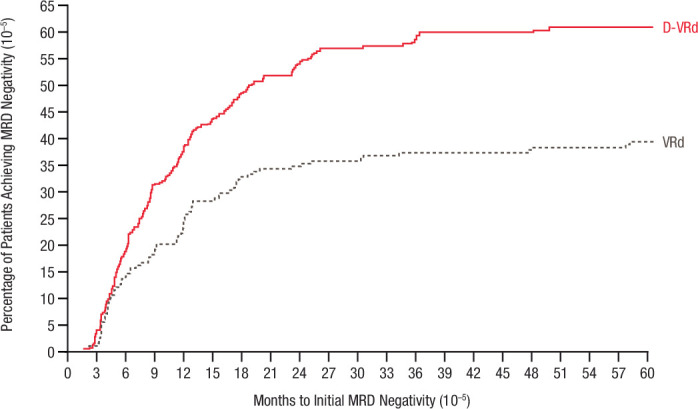
Cumulative incidence of MRD negativity. Shown are the results of the cumulative incidence of MRD negativity. The overall MRD-negativity rate was defined as the proportion of patients who achieved complete response or better and MRD negativity (at or below a sensitivity threshold of 10^–5^) after randomization but prior to disease progression, subsequent antimyeloma therapy or both. D-VRd, subcutaneous daratumumab plus bortezomib/lenalidomide/dexamethasone; MRD, minimal residual disease; VRd, bortezomib/lenalidomide/dexamethasone.

With a median follow-up of 58.7 months (range 0.1–64.7 months), the overall minimal residual disease (MRD)-negativity rate (MRD-negative status (10^−^^5^) with complete response or better (≥CR)) was significantly higher with D-VRd versus VRd (60.9% versus 39.4%; odds ratio, 2.37; 95% confidence interval (CI), 1.58–3.55; *P* < 0.0001; Fig. 2a). The treatment effect on overall MRD-negativity rates was generally consistent across prespecified subgroups (Extended Data Fig. 1). The MRD-negativity rate at 10^−^^6^ was also higher with D-VRd versus VRd (46.2% versus 27.3%; odds ratio, 2.24; 95% CI, 1.48–3.40; *P* = 0.0001; Fig. 2b). Sustained MRD-negativity rate (≥12 months) was significantly higher with D-VRd versus VRd (48.7% versus 26.3%; odds ratio, 2.63; 95% CI, 1.73–4.00; *P* < 0.0001; Fig. 2c). The cumulative incidence of MRD negativity is shown in Extended Data Fig. 2.

**Fig. 3:**
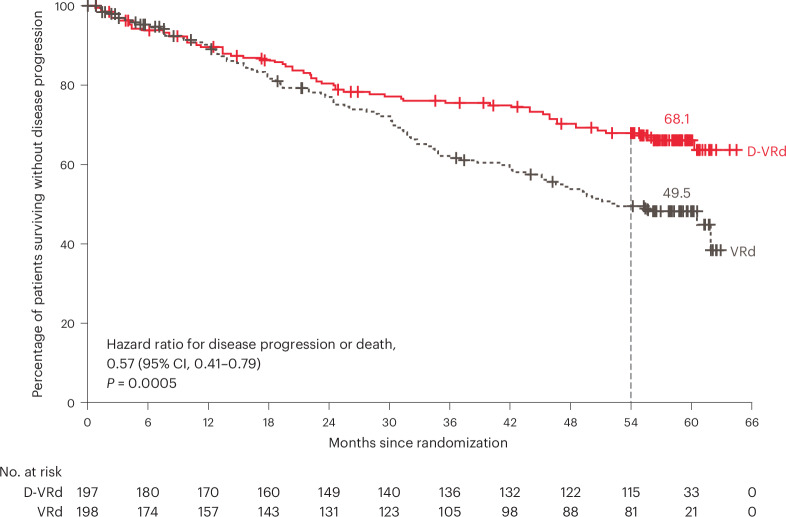
PFS. The results of the Kaplan–Meier estimates of PFS among patients in the intention-to-treat population. The final analysis of PFS was performed after 162 events of disease progression or death occurred. Of these, eight were censored as a result of missing two or more consecutive disease evaluations preceding the event. The *P* value was calculated using the stratified log(rank test).

**Extended Data Fig. 3:**
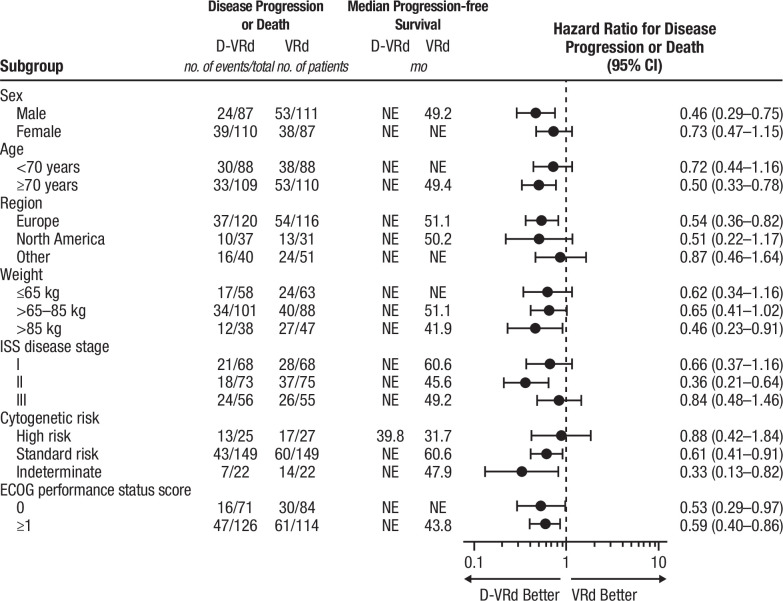
Prespecified subgroup analysis of progression-free survival. Shown are the results of an analysis of progression-free survival in prespecified subgroups in the intention-to-treat population. Data are presented as hazard ratios and 95% CIs. The ISS consists of three stages, with higher stages indicating more severe disease: stage I, serum β_2_-microglobulin level <3.5 mg per liter (300 nmol per liter) and albumin level ≥3.5 g per deciliter; stage II, neither stage I or III; and stage III, serum β_2_-microglobulin level ≥5.5 mg per liter (≥470 nmol per liter). Cytogenetic risk was assessed by fluorescence in situ hybridization. High risk was defined as the presence of del(17p), t(4;14) and/or t(14;16). Indeterminate includes patients with missing or unevaluable samples. CI, confidence interval; D-VRd, subcutaneous daratumumab plus bortezomib/lenalidomide/dexamethasone. ECOG, Eastern Cooperative Oncology Group; ISS, International Staging System; NE, could not be estimated; VRd, bortezomib/lenalidomide/dexamethasone.

Disease progression or death had occurred in 63 patients (32.0%) in the D-VRd group and 91 (46.0%) in the VRd group. D-VRd significantly improved PFS compared with VRd, with a hazard ratio of 0.57 (95% CI, 0.41–0.79; *P* = 0.0005; Fig. 3). Median PFS was not reached for D-VRd versus 52.6 months for VRd; the estimated 54-month PFS rates were 68.1% (95% CI, 60.8–74.3) versus 49.5% (95% CI, 41.8–56.8), respectively. The treatment effect of PFS was generally consistent across the prespecified subgroups (Extended Data Fig. 3).

**Fig. 4:**
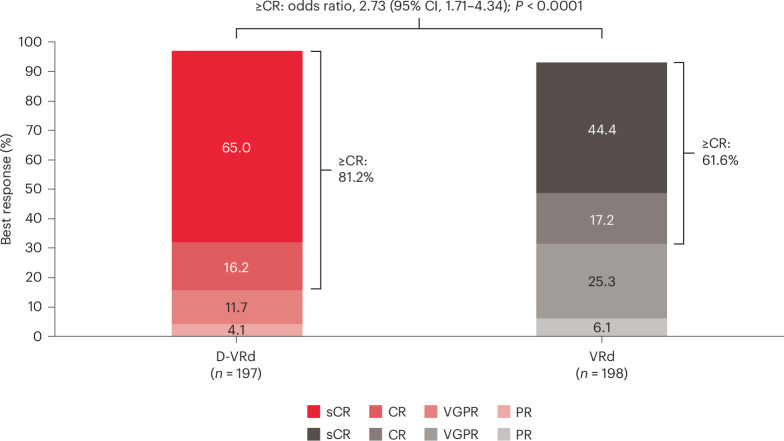
≥CR rates. ≥CR rates in the intention-to-treat population. The tumor response was assessed using a validated computer algorithm in accordance with the International Myeloma Working Group response criteria^26^. Complete response (CR) or stringent CR (sCR) was achieved at any time during the trial. The Mantel–Haenszel estimate of the common odds ratio for stratified tables was used. The stratification factors were ISS disease stage (I, II or III) and age/transplant eligibility (<70 years and transplant ineligible, <70 years and transplant deferred or ≥70 years). An odds ratio >1 indicates an advantage for D-VRd. The *P* value (two sided) was calculated using the Cochran–Mantel–Haenszel *χ*^2^ test. PR, partial response; VGPR, very good partial response.

The ≥CR rate was significantly higher with D-VRd versus VRd (81.2% versus 61.6%; odds ratio, 2.73; 95% CI, 1.71–4.34; *P* < 0.0001; Fig. 4). Additional response data are available in Extended Data Table 2.

**Extended Data Fig. 4:**
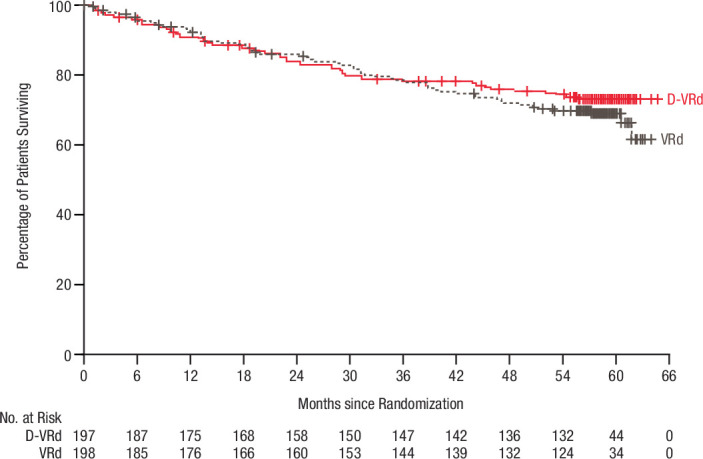
Overall survival. Shown are the results of the Kaplan–Meier estimates of overall survival among patients in the intention-to-treat population. D-VRd, subcutaneous daratumumab plus bortezomib/lenalidomide/dexamethasone; VRd, bortezomib/lenalidomide/dexamethasone.

**Extended Data Fig. 5:**
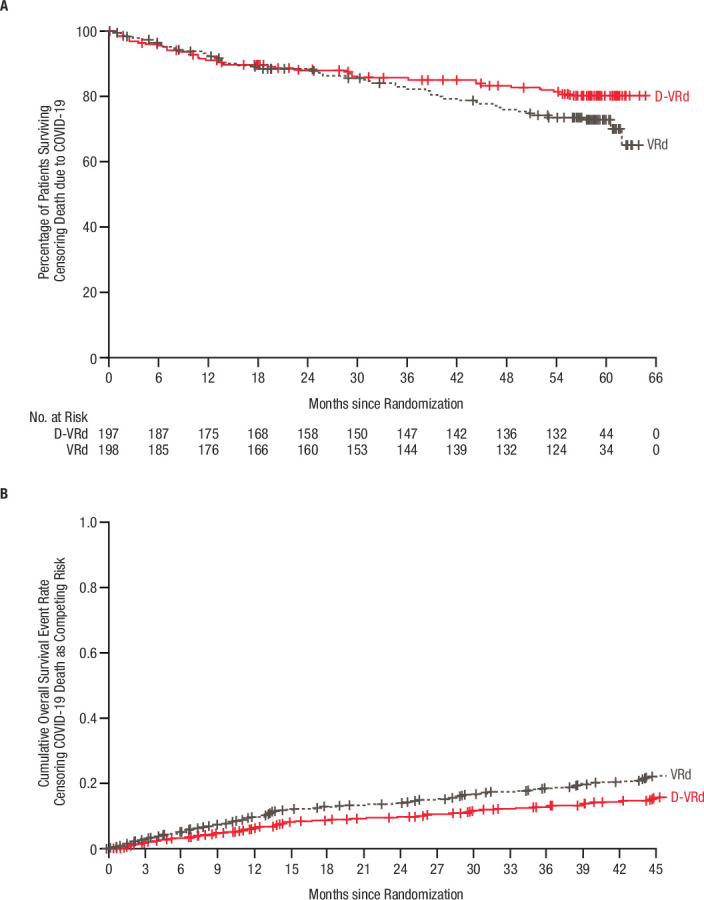
Overall survival censoring death due to COVID-19. Panel A censors death due to COVID-19, and Panel B considers COVID-19 as a competing risk (ie, considers COVID death as a competing event and non-COVID death as the event of interest). D-VRd, subcutaneous daratumumab plus bortezomib/lenalidomide/dexamethasone; VRd, bortezomib/lenalidomide/dexamethasone.

The overall survival hazard ratio trended in favor of D-VRd versus VRd (hazard ratio, 0.85; 95% CI, 0.58–1.24; Extended Data Fig. 4). Overall survival was immature, and follow-up is ongoing. Overall, 51 patients in the D-VRd group and 60 patients in the VRd group died (Extended Data Table 3). The COVID-19 pandemic impacted overall survival in the present study. There were 24 total deaths caused by COVID-19 (21.6% of all deaths on study; 15 for D-VRd and 9 for VRd), 21 of which occurred during the peak of the global pandemic fatalities in 2020 and 2021, with only 3 more occurring in 2022 (after availability of the COVID-19 vaccines) and none in 2023 or 2024 (Supplementary Table 2). Regional variation was observed in countries highly impacted by the pandemic, with the most COVID-19 deaths occurring in Brazil (54.2% of total COVID-19 deaths; 17.5% of study patients recruited) and Poland (16.7% of total COVID-19 deaths; 18.7% of study patients recruited). Two sensitivity analyses of overall survival that adjusted for the impact of COVID-19 deaths showed a more pronounced treatment effect for D-VRd versus VRd: censoring any death caused by COVID-19 (hazard ratio, 0.69; 95% CI, 0.45–1.05) and considering COVID-19 death as a competing risk (hazard ratio for non-COVID mortality, 0.67; 95% CI, 0.44–1.03; Extended Data Fig. 5).

Data for PFS on the next line of therapy (PFS2) are immature; the hazard ratio also favored D-VRd versus VRd (hazard ratio, 0.78; 95% CI, 0.54–1.14; Supplementary Fig. 1). A sensitivity analysis of PFS2 censoring death owing to COVID-19 demonstrated a further improved outcome with D-VRd (hazard ratio, 0.60; 95% CI, 0.40–0.93; Supplementary Fig. 1). A higher proportion of patients who received subsequent therapy received an anti-CD38-based subsequent therapy in the VRd group (39 of 65 patients (60.0%)) than in the D-VRd group (3 of 22 patients (13.6%)).

**Extended Data Fig. 6:**
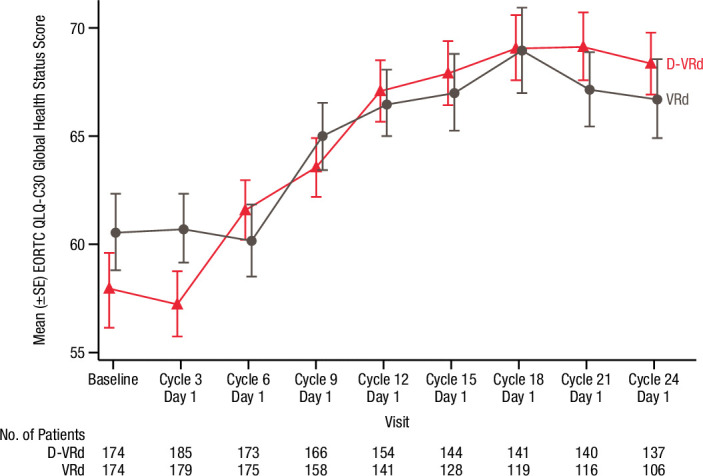
Quality of life. Shown are the results of quality of life in the intention-to-treat population based on the EORTC QLQ-C30 global health status domain score. Data are presented as mean values ± SE. Scores range from 0–100, with higher scores indicating a better health status and quality of life. D-VRd, subcutaneous daratumumab plus bortezomib/ lenalidomide/dexamethasone; EORTC QLQ-C30, European Organisation for Research and Treatment of Cancer Quality of Life Questionnaire–Core 30; SE, standard error; VRd, bortezomib/lenalidomide/dexamethasone.

The European Organisation for Research and Treatment of Cancer Quality of Life Questionnaire–Core 30 (EORTC QLQ-C30) global health domain score improved over time in both groups, with no negative impact from the addition of daratumumab (Extended Data Fig. 6).

### Safety

The most common treatment-emergent adverse events (TEAEs) of any grade (≥20% of patients in either group) and the most common grade 3 or 4 TEAEs (≥10% of patients in either group) are shown in Table 2. The most common grade 3 or 4 TEAEs were neutropenia (44.2% for D-VRd and 29.7% for VRd) and thrombocytopenia (28.4% and 20.0%, respectively). Peripheral neuropathies (peripheral sensory neuropathy, peripheral motor neuropathy, peripheral sensorimotor neuropathy, neuropathy peripheral and/or polyneuropathy) of any grade occurred in 61.9% of patients in the D-VRd group and 66.2% in the VRd group; grade 2 peripheral neuropathy occurred in 31.5% and 36.9% and grade 3 or 4 peripheral neuropathy in 11.2% and 10.8%, respectively.

**Table 2:** Most common adverse events during treatment in the safety population^a^

Event	D-VRd (*n* = 197)		VRd (*n* = 195)	
	Any grade	Grade 3 or 4	Any grade	Grade 3 or 4
Hematological adverse events, no. (%)				
Neutropenia	110 (55.8)	87 (44.2)	76 (39.0)	58 (29.7)
Thrombocytopenia	92 (46.7)	56 (28.4)	66 (33.8)	39 (20.0)
Anemia	73 (37.1)	26 (13.2)	62 (31.8)	23 (11.8)
Lymphopenia	36 (18.3)	24 (12.2)	34 (17.4)	20 (10.3)
Nonhematological adverse events, no. (%)				
Diarrhea	112 (56.9)	24 (12.2)	115 (59.0)	18 (9.2)
Peripheral sensory neuropathy	110 (55.8)	16 (8.1)	119 (61.0)	16 (8.2)
Peripheral edema	83 (42.1)	4 (2.0)	76 (39.0)	1 (0.5)
Constipation	75 (38.1)	4 (2.0)	82 (42.1)	5 (2.6)
Insomnia	63 (32.0)	4 (2.0)	63 (32.3)	2 (1.0)
Fatigue	63 (32.0)	18 (9.1)	60 (30.8)	16 (8.2)
Hypokalemia	58 (29.4)	24 (12.2)	25 (12.8)	12 (6.2)
Cataract	55 (27.9)	17 (8.6)	51 (26.2)	17 (8.7)
Back pain	55 (27.9)	6 (3.0)	43 (22.1)	6 (3.1)
Cough	53 (26.9)	1 (0.5)	38 (19.5)	2 (1.0)
Asthenia	51 (25.9)	7 (3.6)	40 (20.5)	5 (2.6)
Rash	50 (25.4)	5 (2.5)	48 (24.6)	3 (1.5)
Nausea	49 (24.9)	0	48 (24.6)	4 (2.1)
Pyrexia	46 (23.4)	2 (1.0)	30 (15.4)	1 (0.5)
Arthralgia	45 (22.8)	3 (1.5)	39 (20.0)	0
Decreased appetite	42 (21.3)	2 (1.0)	39 (20.0)	5 (2.6)
Dizziness	41 (20.8)	1 (0.5)	41 (21.0)	2 (1.0)
Infection	181 (91.9)	79 (40.1)	167 (85.6)	62 (31.8)
Upper respiratory tract infection	78 (39.6)	1 (0.5)	64 (32.8)	1 (0.5)
COVID-19	75 (38.1)	22 (11.2)	48 (24.6)	9 (4.6)
Pneumonia	48 (24.4)	28 (14.2)	39 (20.0)	25 (12.8)
Urinary tract infection	41 (20.8)	7 (3.6)	29 (14.9)	5 (2.6)
Second primary malignancy, no. (%)	15 (7.6)	N/A	18 (9.2)	N/A
Any injection-related reaction, no. (%)	7 (3.6)	1 (0.5)^b^	N/A	N/A

N/A, not applicable.

^a^The safety population included patients who received ≥1 dose of study treatment. Adverse events of any grade that were reported in ≥20% of patients in either treatment group and grade 3 or 4 adverse events that were reported in at least 10% of patients in either treatment group are listed.

^b^Grade 3.

Serious TEAEs occurred in 72.1% of patients in the D-VRd group and 67.2% in the VRd group (Extended Data Table 4). The most common serious TEAE was pneumonia (D-VRd, 13.7%; VRd, 12.8%). The rate of treatment discontinuation caused by TEAEs was 7.6% for D-VRd and 15.9% for VRd. Discontinuation rates and treatment modifications by individual study drug are included in Supplementary Table 3. Discontinuations and dose modifications of all or any study treatment and of bortezomib specifically caused by peripheral neuropathy were similar between the groups (Supplementary Table 4). Non-COVID-related and COVID-related grade 5 TEAEs occurred in 10.7% and 6.1% of patients, respectively, in the D-VRd group and 7.7% and 3.1% of patients, respectively, in the VRd group. Most grade 5 events occurred after discontinuation of bortezomib (cycle 8) in both arms (13% D-VRd versus 9% VRd). When adjusted for treatment exposure, the rate of grade 5 TEAEs was comparable between groups (D-VRd, 0.39 out of 100 patient-months; VRd, 0.31 out of 100 patient-months). Second primary malignancies were observed in 15 patients (7.6%) in the D-VRd group and 18 patients (9.2%) in the VRd group (Supplementary Table 5). Of all second primary malignancies, cutaneous malignancies represented 7 (3.6%) patients in the D-VRd group and 7 (3.6%) in the VRd group.

## Discussion

Results from this final PFS analysis of CEPHEUS, with a median follow-up of 58.7 months, demonstrated that combining daratumumab with VRd significantly improved clinical outcomes, including overall MRD negativity and PFS, versus VRd alone in patients with transplant-ineligible or transplant-deferred NDMM. The deeper responses achieved with D-VRd translated into a superior PFS, with a significant 43% lower risk of disease progression or death. Overall survival data, although immature, showed a trend favoring D-VRd. Sensitivity analyses provided stronger evidence of the treatment effect of D-VRd on overall survival after adjusting for the impact of COVID-19.

Triplet therapy such as D-Rd is the standard of care for patients who are transplant ineligible in many countries; therefore, it is important to look at the tolerability of adding bortezomib to the D-VRd regimen. In the phase 3 ALCYONE study, adding daratumumab to bortezomib, melphalan and prednisone did not increase overall toxicity versus triplet therapy alone, and the incidence of peripheral neuropathy was lower in the daratumumab group^18^. The most common grade 3 or 4 TEAEs in CEPHEUS were neutropenia and thrombocytopenia. The incidence of grade 2 peripheral neuropathy was lower with D-VRd versus VRd, and the incidence of grade 3 or 4 peripheral neuropathy was similar between the groups. The incidence of grade 5 TEAEs was higher in the D-VRd group, as the result of more grade 5 COVID-19 events and nearly 2 years of additional treatment exposure in the D-VRd arm. With most grade 5 TEAEs occurring after bortezomib discontinuation, the higher incidence of these events with D-VRd reflects the prolonged treatment exposure. When adjusted for exposure, taking into account the almost 2 years of additional treatment received in the D-VRd arm, the rate of grade 5 TEAEs was comparable between groups. The incidence of second primary malignancies was lower in the D-VRd group.

Cross-trial comparisons should be interpreted with caution due to differences in patient populations and trial designs but can help to contextualize our findings. Before data availability from CEPHEUS, results from the MAIA study established D-Rd as a standard of care for transplant-ineligible patients, with a median overall survival of 7.5 years and consistent benefit across age, fitness and risk status subgroups^8,13,14,19^. It is important to note that all patients enrolled in MAIA were transplant ineligible, the population included frail patients and there was no upper age limit (~19% were aged ≥80 years)^19^, whereas CEPHEUS enrolled transplant-ineligible and transplant-deferred patients, excluded frail patients and no patients were aged >80 years. Although the PFS benefit observed in MAIA was impressive, particularly considering the inclusion of frail and older patients, quadruplet therapy with D-VRd offers an opportunity for improved depth of response, with estimated 48-month PFS rates of 59.4% for D-Rd in MAIA^20^ and 70.4% for D-VRd in CEPHEUS. With D-VRd, physicians have an increased ability to tailor frontline daratumumab-based combination therapy to the patient’s age, frailty and other patient-related and disease-related risk factors. It will be important to balance the higher depth of response and longer PFS achievable with quadruplet D-VRd therapy with the improved tolerability offered by triplet D-Rd therapy, with the ultimate decision probably based on the individual patient’s overall treatment goals and perceived ability to tolerate the addition of bortezomib.

The phase 3 IMROZ study evaluated intravenous isatuximab plus VRd versus VRd alone in transplant-ineligible patients with NDMM^21^. The CEPHEUS patient population included both transplant-ineligible and transplant-deferred patients. The median age was similar in CEPHEUS (70 years) and IMROZ (72 years), and a similar proportion of patients had high cytogenetic risk (13.2% and 16.6%, respectively). At a median follow-up of 59.7 months, the hazard ratio for disease progression or death was 0.60 (95% CI, 0.41–0.88; *P* < 0.001) for IMROZ versus 0.57 (95% CI, 0.41–0.79; *P* = 0.0005) for CEPHEUS (median follow-up: 58.7 months)^21^. The median PFS for VRd was similar in both studies (54.3 months in IMROZ and 52.6 months in CEPHEUS). MRD-negativity rates (55.5% versus 40.9%; *P* = 0.003) and ≥CR rates (74.7% versus 64.1%; *P* = 0.01) were higher with isatuximab plus VRd versus VRd alone^21^. As expected, the incidence of grade 5 TEAEs was higher with quadruplet versus triplet therapy (approximately twice as high in both studies). The incidence of infusion- or injection-related reactions was considerably higher with intravenous isatuximab in IMROZ (23.6%) compared with subcutaneous daratumumab in CEPHEUS (3.6%). There were differences in terms of timing of study enrollment, with CEPHEUS being initiated later relative to the start of the COVID-19 pandemic (December 2018 versus December 2017 for IMROZ), meaning that more patients in CEPHEUS were likely to still be on study treatment and at risk of COVID-19 infection during the pandemic compared with IMROZ.

Use of quadruplet therapy was also recently reported in the transplant-eligible population. The phase 3 PERSEUS study showed significant and clinically meaningful benefit in terms of PFS (hazard ratio, 0.42; *P* < 0.001), ≥CR rate (87.9% versus 70.1%; *P* < 0.001) and MRD-negativity rate (75.2% versus 47.5%; *P* < 0.001) with D-VRd induction/consolidation followed by daratumumab–lenalidomide maintenance versus VRd induction/consolidation and lenalidomide maintenance alone^15^. Taken together, these data from CEPHEUS, combined with the results from MAIA^8,13,14^ and PERSEUS^15^, further demonstrate the important role of daratumumab-based triplet and quadruplet therapy in deepening and prolonging responses for all patients across the frontline treatment spectrum. Many older patients with NDMM may not receive subsequent therapy^22–24^, highlighting the importance of choosing the most effective regimen in the first-line setting. Frontline quadruplet D-VRd therapy provides an opportunity to further deepen responses and improve clinical outcomes.

A potential limitation of the CEPHEUS study is that patients who are Black or African American represented 4.8% of the total study population, which in some countries may be an underrepresentation. However, this trial was partly conducted in countries where race was not reported based on trial regulations. Race ‘not reported’ represented 7.3% of the study population.

In conclusion, with almost 5 years of follow-up, results from CEPHEUS show that the addition of daratumumab to VRd significantly increased depth of response, including rates of overall MRD negativity, ≥CR and sustained MRD negativity, which translated to significantly improved PFS versus VRd alone in transplant-ineligible or transplant-deferred patients with NDMM. Moreover, our results add further validity to the use of MRD negativity as an accelerated approval endpoint to predict PFS outcomes in NDMM. The safety profile of D-VRd was consistent with that of each individual agent. These data, together with the phase 3 PERSEUS study, demonstrate the consistent benefit of quadruplet daratumumab plus VRd therapy compared with triplet VRd therapy and support D-VRd quadruplet therapy as a new standard of care for NDMM, regardless of transplant eligibility.

## Methods

### Trial design and oversight

This randomized, open-label, multicenter, phase 3 study enrolled patients between 11 December 2018 and 7 October 2019 at 92 sites in 13 countries (Supplementary Information). Patients were randomly assigned (1:1) to D-VRd or VRd by randomly permuted blocks using an interactive web-response system. Randomization was stratified by ISS disease stage (I, II or III) and age or transplant eligibility (<70 years and transplant ineligible, <70 years and transplant deferred or ≥70 years). There was no selection of patients.

### Inclusion and ethics

An independent ethics committee or institutional review board approved the protocol at each site. The study was conducted in accordance with the International Conference on Harmonisation Good Clinical Practice guidelines, the principles originating from the Declaration of Helsinki and study site-specific regulations. All patients provided written informed consent.

### Patients

Enrolled patients had NDMM^27^, an ECOG performance status score of 0–2 and a frailty index <2 (ref. ^28^), were either aged <80 years and not considered candidates for high-dose chemotherapy with stem-cell transplantation because of their age (≥70 years) or aged 18–70 years with the presence of underlying medical conditions likely to have a negative impact on tolerability of high-dose chemotherapy with stem-cell transplantation, making them transplant ineligible or refusing high-dose chemotherapy with stem-cell transplantation as the initial treatment (transplant deferred). Sex was collected and reported in the trial; sex was reported by the patient. Patients had an absolute neutrophil count of ≥1.0 × 10^9^ l^−1^ (granulocyte–colony-stimulating factor was permitted), a hemoglobin level of ≥7.5 g dl^−1^ (without prior red blood cell transfusion within 7 d before the laboratory test; recombinant human erythropoietin use was permitted), a platelet count of ≥70 × 10^9^ l^−1^ (if <50% of bone marrow-nucleated cells were plasma cells; otherwise, the platelet count was >50 × 10^9^ l^−1^), a calculated creatinine clearance of ≥30 ml min^−1^, a corrected serum calcium level of ≤13.5 mg dl^−1^ (≤3.4 mmol l^−1^) or free ionized calcium level of ≤6.5 mg dl^−1^ (≤1.6 mmol l^−1^), aspartate and alanine aminotransferase levels ≤2.5 times the upper limit of normal and a total bilirubin level ≤1.5 times the upper limit of normal. Excluded were patients with prior therapy for multiple myeloma other than a short course of corticosteroids, prior or concurrent invasive malignancy (other than multiple myeloma) within 5 years of randomization, grade ≥2 peripheral neuropathy or neuropathic pain (per National Cancer Institute Common Terminology Criteria for Adverse Events (NCI-CTCAE), v.5), focal radiotherapy within 14 d of randomization, plasmapheresis within 28 d of randomization, clinical signs of meningeal involvement of multiple myeloma, chronic obstructive pulmonary disease with a forced expiratory volume in 1 s <50% of predicted normal, moderate or severe persistent asthma within the past 2 years or currently uncontrolled asthma.

### Trial treatments

All patients received eight 21-day cycles of VRd, consisting of subcutaneous bortezomib (1.3 mg m^−2^ on days 1, 4, 8 and 11), oral lenalidomide (25 mg on days 1–14) and oral or intravenous dexamethasone (20 mg on days 1, 2, 4, 5, 8, 9, 11 and 12 (days 1, 4, 8 and 11 if aged >75 years or body mass index <18.5 kg m^−2^)), after which point bortezomib was discontinued per protocol and patients continued to receive 28-d cycles of Rd, consisting of oral lenalidomide (25 mg on days 1–21) and oral dexamethasone (40 mg on days 1, 8, 15 and 22 (20 mg weekly if aged >75 years or body mass index <18.5 kg m^−2^)) until progression or unacceptable toxicity. Patients in the D-VRd group also received subcutaneous daratumumab (daratumumab 1,800 mg co-formulated with recombinant human hyaluronidase PH20 (2,000 U ml^−1^; ENHANZE drug delivery technology, Halozyme, Inc.)) weekly in cycles 1–2, every 3 weeks in cycles 3–8 and every 4 weeks thereafter until progression or unacceptable toxicity. Supplementary Information includes details about pre- and post-administration medications.

### Endpoints and assessments

The primary endpoint was the overall MRD-negativity rate, defined as the proportion of patients who achieved ≥CR and had MRD-negative status (at or below a sensitivity threshold of 10^–5^) after randomization but before progression, subsequent antimyeloma therapy or both. Major secondary endpoints were ≥CR rate, PFS and sustained MRD-negativity rate (≥12 months). Secondary endpoints are defined in Supplementary Information.

MRD was evaluated via next-generation sequencing using the clonoSEQ assay with bone marrow aspirate samples obtained at baseline, at the time of suspected CR and at 12, 18, 24, 30 and 36 months after the first dose and annually thereafter in patients who achieved a confirmed CR. Tumor response and disease progression were assessed using a validated computer algorithm in accordance with International Myeloma Working Group response criteria 2011 (ref. ^26^). Disease assessments were performed at a central laboratory. TEAEs were graded according to the NCI-CTCAE v.5. TEAEs were reported until 30 d after the last dose of any component of the treatment regimen.

### Statistical analysis

We estimated that a sample size of 360 patients (180 in each arm) was needed to achieve 80% power to detect a 15% treatment difference in overall MRD-negativity rate at a two-sided alpha of 0.05. This sample size would also provide 80% power to detect a 37% reduction in the risk of disease progression or death with a log(rank test) at a two-sided alpha of 0.05. The primary analysis and final analysis (described in this article) were performed in the intention-to-treat population, which included all randomized patients. The safety population included all patients who received ≥1 dose of the assigned treatment.

If the primary endpoint of the overall MRD-negativity rate was statistically significant, the major secondary endpoints (that is, ≥CR rate, PFS and sustained MRD-negativity rate) were sequentially tested, each with an overall two-sided *α* of 0.05, using a hierarchical testing approach as proposed by Tang and Geller^29^ that strongly controls the family-wise type I error rate. Overall MRD-negativity rates and rates of ≥CR were compared between groups using the stratified Cochran–Mantel–Haenszel test. Time-to-event endpoints, including PFS, were compared between groups using a stratified log(rank test). Hazard ratios and 95% CIs were estimated using a stratified Cox regression model with treatment as the sole explanatory variable, stratified by ISS disease stage (I, II or III) and age/transplant eligibility (<70 years and transplant ineligible, <70 years and transplant deferred or ≥70 years). Landmark PFS rates and 95% CIs were estimated using the Kaplan–Meier method.

The primary analysis of MRD was performed approximately 18 months after the last patient was administered the first study treatment dose. An interim analysis of PFS was planned when approximately 98 algorithm-based PFS events (60% of the total planned events) had been accumulated. At both the primary MRD analysis and interim PFS analysis, the Independent Data Monitoring Committee recommended continuing the study unmodified and the sponsor remained blinded. The final PFS analysis occurred when approximately 162 algorithm-based PFS events had been reached, after which point the study was routinely unblinded. We report results for this final PFS analysis.

### Reporting summary

Further information on research design is available in the Nature Portfolio Reporting Summary linked to this article.

## Online content

Any methods, additional references, Nature Portfolio reporting summaries, source data, extended data, supplementary information, acknowledgements, peer review information; details of author contributions and competing interests; and statements of data and code availability are available at 10.1038/s41591-024-03485-7.

## Supplementary information

Supplementary Information Collaborators, Study sites, Additional methods, Supplementary Fig. 1, Tables 1–5 and References.

Reporting Summary

## Figures and Tables

**Extended Data Table 1:**
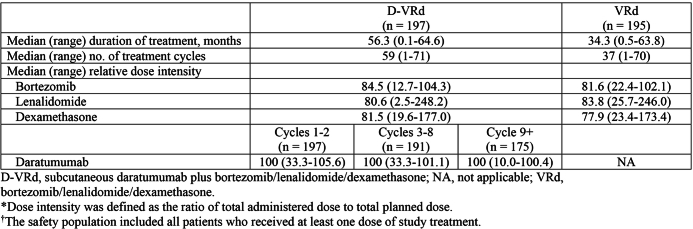
Duration of treatment and relative dose intensities* in the safety population^†^

**Extended Data Table 2:**
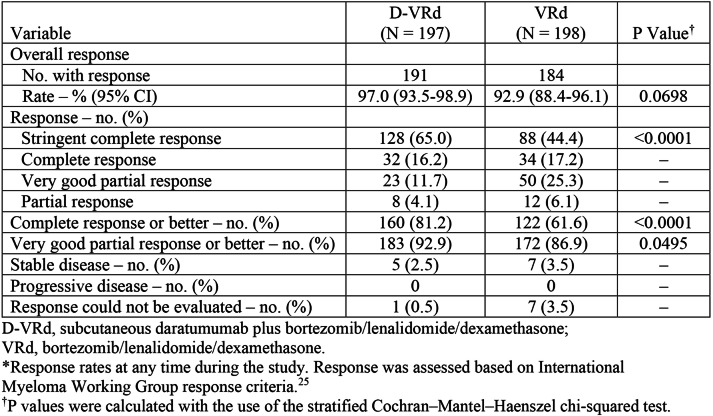
Summary of response rates in the intention-to-treat population*

**Extended Data Table 3:**
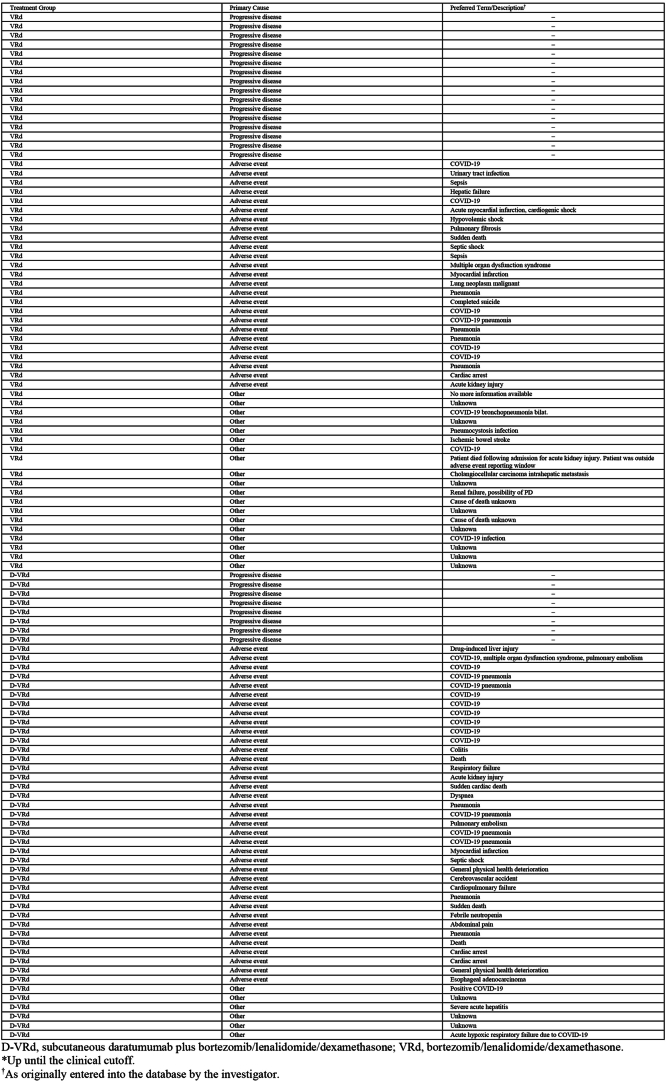
List of all deaths on study* in the intention-to-treat population

**Extended Data Table 4:**
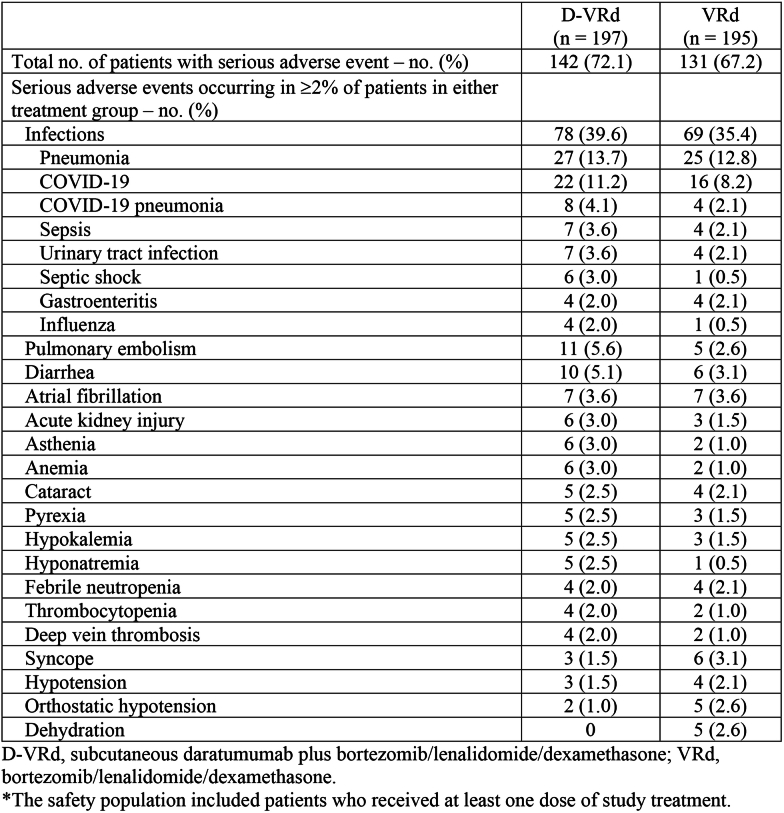
Serious adverse events in the safety population*

## Data Availability

The data sharing policy of Johnson & Johnson is available at https://innovativemedicine.jnj.com/our-innovation/clinical-trials/transparency. As noted on this site, requests for access to the study data can be submitted through the Yale Open Data Access (YODA) Project site at http://yoda.yale.edu. The trial protocol and statistical analysis plan can be found in the Supplementary Information.

## Code availability

The CEPHEUS study used electronic Case Report Forms to collect data from the participants (v.9.00, 08JUN2022 MH). All statistical analyses were performed using SAS Software 9.4 (TS1M6 MBCS3170).
