# Author Correction: Daratumumab plus bortezomib, lenalidomide and dexamethasone for transplant-ineligible or transplant-deferred newly diagnosed multiple myeloma: the randomized phase 3 CEPHEUS trial

**DOI:** 10.1038/s41591-025-03581-2

**Published:** 2025-02-13

**Authors:** Saad Z. Usmani, Thierry Facon, Vania Hungria, Nizar J. Bahlis, Christopher P. Venner, Marc Braunstein, Ludek Pour, Josep M. Martí, Supratik Basu, Yael C. Cohen, Morio Matsumoto, Kenshi Suzuki, Cyrille Hulin, Sebastian Grosicki, Wojciech Legiec, Meral Beksac, Angelo Maiolino, Hiroyuki Takamatsu, Aurore Perrot, Mehmet Turgut, Tahamtan Ahmadi, Weiping Liu, Jianping Wang, Katherine Chastain, Jessica Vermeulen, Maria Krevvata, Lorena Lopez-Masi, Jodi Carey, Melissa Rowe, Robin Carson, Sonja Zweegman

**Affiliations:** 1 Memorial Sloan Kettering Cancer Center, New York, NY USA; 2 University of Lille, CHU de Lille, Service des Maladies du Sang, Lille, France; 3 Clínica Médica São Germano, São Paulo, Brazil; 4 Arnie Charbonneau Cancer Research Institute, University of Calgary, Calgary, Alberta Canada; 5 Department of Medical Oncology, Cross Cancer Institute, University of Alberta, Edmonton, Alberta Canada; 6 BC Cancer—Vancouver Centre, University of British Columbia, Vancouver, British Columbia Canada; 7 Perlmutter Cancer Center, NYU Langone Health, New York, NY USA; 8 University Hospital Brno, Brno, Czech Republic; 9 Hospital Universitario Mútua de Terrassa, Terrassa, Spain; 10 Royal Wolverhampton NHS Trust and University of Wolverhampton, CRN West Midlands, National Institute for Health and Care Research, Wolverhampton, UK; 11 Department of Hematology, Tel Aviv Sourasky (Ichilov) Medical Center, Tel Aviv, Israel; 12 Faculty of Medical and Health Sciences, Tel Aviv University, Tel Aviv, Israel; 13 Department of Hematology, National Hospital Organization Shibukawa Medical Center, Gunma, Japan; 14 Department of Hematology, Japanese Red Cross Medical Center, Tokyo, Japan; 15 Department of Hematology, Hôpital Haut Lévêque, University Hospital, Pessac, France; 16 Department of Hematology and Cancer Prevention, School of Public Health, Medical University of Silesia, Katowice, Poland; 17 Department of Hematology and Bone Marrow Transplantation, St. John of Dukla Oncology Center of Lublin Land, Lublin, Poland; 18 Istinye University, Ankara Liv Hospital, Ankara, Turkey; 19 Instituto Americas de Ensino, Pesquisa e Inovação, Rio de Janeiro, Brazil; 20 Universidade Federal do Rio de Janeiro, Rio de Janeiro, Brazil; 21 Department of Hematology, Kanazawa University Hospital, Kanazawa University, Kanazawa, Japan; 22 CHU de Toulouse, IUCT-O, Université de Toulouse, UPS, Service d’Hématologie, Toulouse, France; 23 Department of Hematology, Ondokuz Mayıs University Faculty of Medicine, Samsun, Turkey; 24 Genmab US, Inc., Plainsboro, NJ USA; 25 Johnson & Johnson, Shanghai, China; 26 Johnson & Johnson, Spring House, PA USA; 27 Johnson & Johnson, Raritan, NJ USA; 28 Johnson & Johnson, Leiden, The Netherlands; 29 Johnson & Johnson, High Wycombe, UK; 30 Department of Hematology, Amsterdam UMC, Vrije Universiteit Amsterdam, Cancer Center Amsterdam, Amsterdam, The Netherlands

**Keywords:** Myeloma, Targeted therapies

Correction to: *Nature Medicine* 10.1038/s41591-024-03485-7, published online 5 February 2025.

In the version of the article initially published, the title read “…phase 3 CEPHEUS study” rather than “…phase 3 CEPHEUS trial” and has now been corrected in the HTML and PDF versions of the article.

