# Supplementary material for: Daratumumab plus bortezomib, lenalidomide and dexamethasone for transplant-ineligible or transplant-deferred newly diagnosed multiple myeloma: the randomized phase 3 CEPHEUS trial
Source: Nat Med. 2025 Feb 5;31(4):1195–202. doi: 10.1038/s41591-024-03485-7 (PMC12003169; doi:10.1038/s41591-024-03485-7)
Supplement: Supplementary file 1 — Collaborators, Study sites, Additional methods, Supplementary Fig. 1, Tables 1–5 and References. [file 41591_2024_3485_MOESM1_ESM.pdf]

nature medicine

Supplementary information https://doi.org/10.1038/s41591-024-03485-7

Daratumumab plus bortezomib, lenalidomide and dexamethasone for transplant-ineligible or transplant-deferred newly diagnosed multiple myeloma: the randomized phase 3 CEPHEUS trial

In the format provided by the authors and unedited

Table of Contents

Collaborators 2

Study Sites 4

Additional Methods 6

Supplementary Fig. 1. Progression-free Survival on Next Line of Therapy (PFS2). 10

Supplementary Table 1. Study Enrollment by Region and Country in the Intention-to-Treat Population.* 12

Supplementary Table 2. Summary of Total COVID-19 Deaths and COVID-19 Serious Adverse Events By Year in the Safety Population.* 13

Supplementary Table 3. Summary of Treatment Modifications and Discontinuations in the Safety Population.* 14

Supplementary Table 4. Summary of Patients With Treatment Discontinuation or Dose Modification Due to Treatment-emergent Peripheral Neuropathy in the Safety Population.* 15

Supplementary Table 5. Summary of Second Primary Malignancies in the Safety Population.* 16

References 17

Collaborators

The following principal investigators participated in the CEPHEUS study:

Brazil: Karin Cecyn, Angelo Maiolino, Rodrigo Santucci Alves Da Silva, Carolina Colaco, Paula Vanessa De Oliveira, Breno Gusmao, Vania Hungria, Fernando Pericole, Mariza Schaan, Reijane Alves de Assis, Luciana Barreto, Juliana Boekel, Marcelo Bellesso, Jandey Bigonha, Marcelo Eduardo Zanella, Edvan Crusoe, James Maciel, Fernanda Seguro, Flavia Xavier, Maria Macedo; **Canada:** Nizar Bahlis, Ariah Schattner, Michael Sebag, Darrell White, Kevin Song, Marc Lalancette, Michael Chu, Martha Louzada, Christopher Venner, Heather Sutherland, Arleigh McCurdy, Philip Kuruvilla; **Czechia:** Ludek Pour, Roman Hajek, Ivan Spicka, Alexandra Jungova, Vladimír Maisnar, Jiri Minarik, Petr Pavlicek; **France:** Aurore Perrot, Frederic Maloisel, Cyrille Hulin, Mourad Tiab, Jean-Marc Schiano De Colella, Karim Belhadj-Merzoug, Laure Vincent, Thierry Facon, Michel Attal, Laurent Garderet, Anne-Marie Stoppa, Philippe Moreau; **Germany:** Britta Besemer, Thomas Illmer, Christian Krogel, Christian Lerchenmueller, Christian Schmidt, Sebastian Theurich, Manfred Welslau, Heinz Duerk, Monika Engelhardt, Hartmut Goldschmidt, Dietger Niederwieser, Wolfram Pönisch, Joerg Thomalla, Katja Weisel, Carsten Bokemeyer; **Israel:** Yael Cohen, Osnat Jarchowsky, Anatoly Nemets, Inna Tzoran, Alon Rosenthal, Orit Sofer, Irit Avivi, Najib Dally, Moshe Gatt, Hila Magen, Meir Preis, Celia Suriu, Tamar Tadmor, Iuliana Vaxman; **Japan:** Morio Matsumoto, Shinsuke Iida, Hiroshi Kosugi, Tomoaki Fujisaki, Kazunori Imada, Tadao Ishida, Toshihiro Miyamoto, Tadakazu Kondo, Kenshi Suzuki, Hiroyuki Takamatsu, Hitomi Kaneko, Takayuki Ishikawa, Toshiro Ito, Junya Kuroda, Kazutaka Sunami, Yasushi Takamatsu, Naoko Harada, Michihiro Hidaka; **Korea:** Soo-Mee Bang, Hyeon Seok Eom, Jin Seok Kim, Kihyun Kim, Jae Hoon Lee, Je-Jung Lee, Chang Ki Min, Sung-Soo Yoon; **Netherlands:** Sonja Zweegman, Mark-David

Levin, Pieter Sonneveld; **Poland:** Magdalena Dutka, Sebastian Grosicki, Wojciech Homenda, Dominik Dytfeld, Pawel Steckiewicz, Wojciech Legiec, Bartosz Pula, Artur Jurczyszyn, Jan Walewski, Jacek Krzanowski, Krzysztof Jamroziak, Andrzej Pluta, Jan Maciej Zaucha; **Spain:** Carmen Martinez-Chamorro, Eugenia Abella Monreal, Isabel Krsnik Castello, Josep Maria Marti Tutusaus, Francisco Javier Penalver Parraga, Dunia de Miguel Llorente, Carmen Aguilera, Rosalía Riaza Grau; **Turkey:** Fevzi Altuntas, Mehmet Turgut, Meltem Ayli, Guldane Cengiz Seval, Sevgi Kalayoglu Besisik, Hakan Goker, Inci Alacacioglu, Meral Beksac, Hayri Ozsan, Can Boga, Tulin Tuglular; **United Kingdom:** Khalid Saja, Feargal McNicholl, Ceri Bygrave, Hannah Mary Hunter, Mark Grey, Iain Singer, Supratik Basu, Mamta Garg, Antonina Zhelyazkova, Lalita Banerjee, Michael Hamblin, Helen McCarthy, Jane Tighe, Catherine D. Williams; **United States:** Daniel Huang, Christy Samaras, Jens Hillengass, Raymond Comenzo, Mansi Shah, Laahn Foster, William Hammond, Joseph Maly, John Strother, Tondre Buck, Marc Braunstein, Sunil Babu, Robert Orlowski, Shebli Atrash, Carl Gray, Roberto Ferro, Asim Pati, Shahzad Raza, Alfred Saleh, Casie Shenoy, Cindy Varga, Maurizio Bendandi, Jennifer Cudris, Salyka Sengsayadeth, Kristen Sanfilippo, Mark Schroeder, Tanya Wildes, Keith Goldstein, Brett Brinker, Peter Byeff, David Calverley, Asher Chanan Khan, Yvonne Efebera, Ashley Rosko, Daphne Friedman, Charles Holladay, Sardar Imam, Shaji Kumar, Jacob Laubach, Ruben Niesvizky, Gregory Kaufman, Terri Parker, Brandi Reeves, Ruben Saez, Vaishali Sanchorawala, Leyla Shune, Roger Strair, Dennis Cooper, Vimal Patel, Saad Usmani, Jason Valent, Natalie Callander, Christopher Fletcher, Anastasios Raptis

Study Sites

The following study sites enrolled at least 1 patient in the CEPHEUS study:

Brazil: Hospital Santa Cruz; Instituto de Educacao, Pesquisa e Gestao em Saude Instituto Americas (COI); Instituto de Ensino e Pesquisa São Lucas; Liga Norte Riograndense Contra O Cancer; Ministerio da Saude - Instituto Nacional do Cancer; Real e Benemerita Associacao Portuguesa de Beneficencia; Clinica Sao Germano; Universidade Estadual De Campinas; Uniao Brasileira de Educacao e Assistencia Hospital Sao Lucas da PUCRS; **Canada:** Tom Baker Cancer Centre; Lakeridge Health Oshawa; McGill University Health Centre; QEII Health Sciences Centre; The Gordon & Leslie Diamond Health Care Center; CHU de Quebec L Hotel Dieu de Quebec; Cross Cancer Institute; Victoria Hospital; **Czechia:** Fakultni nemocnice Brno; Fakultni Nemocnice Ostrava; Vseobecna fakultni nemocnice v Praze; Fakultni nemocnice Plzen, Hemato-onkologicke oddeleni; Fakultni nemocnice Hradec Kralove; **France:** Institut Universitaire du cancer de Toulouse-Oncopole; Strasbourg Oncologie Libérale; CHU de Bordeaux - Hospital Haut-Leveque; Centre Hospitalier Départmental La Roche sur Yon; Institut Paoli Calmettes; CHU Henri Mondor; CHU de Montpellier Hopital Saint Eloi; Hopital Claude Huriez; **Germany:** Universitaetsklinikum Tuebingen der Eberhard-Karls-Universitaet, Abteilung fuer Innere Medizin II; **Israel:** Sourasky (Ichilov) Medical Center; Meir Hospital; Barzilai Medical Center; Rambam Med.Center - Hematology Institute; Rabin Medical Center; Hillel Yaffe Medical Center; **Japan:** National Hospital Organization Shibukawa Medical Center; Nagoya City University Hospital; Ogaki Municipal Hospital; Matsuyama Red Cross Hospital; Japanese Red Cross Osaka Hospital; Japanese Red Cross Medical Center; Kanazawa University Hospital; Kobe City Medical Center General Hospital; **Netherlands:** Amsterdam UMC; Albert Schweitzer ziekenhuis-lokatie Dordwijk; **Poland:** Uniwersyteckie Centrum Kliniczne;

Samodzielny Publiczny Zaklad Opieki Zdrowotnej Zespol Szpitali Miejskich; Wojewodzki Szpital Specjalistyczny im. Janusza Korczaka; Uniwersytecki Szpital Kliniczny w Poznaniu; Swietokrzyskie Centrum Onkologii SPZOZ w Kielcach; Centrum Onkologii Ziemi Lubelskiej im. sw. Jana z Dukli; Instytut Hematologii i Transfuzjologii; NSSU Szpital Uniwersytecki w Krakowie; Narodowy Instytut Onkologii im Marii Sklodowskiej Curie Panstwowy Instytut Badawczy; Szpital Specjalistyczny w Brzozowie Podkarpacki Osrodek Onkologiczny im Ks B Markiewicza; **Spain:** Hosp. Quiron Madrid Pozuelo; Hosp. Del Mar; Hosp. Univ. PTA. De Hierro Majadahonda; Hosp. Mutua Terrassa; Hosp. Univ. Fundacion Alcorcon; **Turkey:** Dr Abdurrahman Yurtaslan Oncology Training and Research Hospital; Ondokuz Mayis Universitesi Tip Fakultesi; Gulhane Egitim ve Arastirma Hastanesi; Ankara University School of Medicine Cebeci Hospital; Istanbul University Istanbul Medical Faculty; Hacettepe University Medical Faculty; Dokuz Eylul University Medical Faculty; **United Kingdom:** Colchester Hospital University NHS; Altnagelvin Hospital; University Hospital Wales; Derriford Hospital; Blackpool Victoria Hospital; Monklands District General Hospital; New Cross Hospital; Leicester Royal Infirmary – Haematology; The Royal Oldham Hospital; **United States:** Innovative Clinical Research Inc; Cleveland Clinic; Roswell Park Cancer Institute; Tufts Medical Center; Rutgers Cancer Institute of New Jersey; University of Virginia; Baptist MD Anderson; Norton Healthcare; Good Samaritan Hospital Corvallis; Gibbs Cancer Center; NYU Langone Hospital—Long Island; Fort Wayne Medical Oncology and Hematology, Inc.; University of Texas, MD Anderson Cancer Center; Levine Cancer Institute;

Additional Methods

Pre- and Post-administration Medications

To decrease the risk of injection-related reactions, all patients in the daratumumab plus bortezomib, lenalidomide and dexamethasone (D-VRd) group received intravenous or oral acetaminophen (650-1000 mg), an antihistamine (intravenous or oral diphenhydramine 25-50 mg or equivalent), and oral montelukast (10 mg, recommended on cycle 1 day 1) only up to 24 hours prior to daratumumab injection.

Patients with mild asthma or chronic obstructive pulmonary disease who have a FEV1 of <80% could receive post-injection medications, including an antihistamine, a short-acting β2 adrenergic receptor agonist (such as salbutamol), and control medications for lung disease (eg, inhaled corticosteroids ± long-acting β2 adrenergic receptor agonists for patients with asthma or long-acting bronchodilators [such as tiotropium or salmeterol] ± inhaled corticosteroids for patients with chronic obstructive pulmonary disease).

Endpoints and Assessments

Overall minimal residual disease (MRD)–negativity rate was defined as the proportion of patients who achieved complete response or better and had MRD-negative status (at or below a sensitivity threshold of 10^{-5}) after randomization but prior to disease progression, subsequent antimyeloma therapy, or both. MRD-positive patients included patients for whom all tested samples were found to be MRD positive or indeterminate. For patients with missing MRD samples, failure to calibrate baseline MRD, or otherwise unevaluable samples, MRD status was considered as MRD positive.

Complete response or better rate was defined as the proportion of patients achieving complete response or stringent complete response based on the computerized algorithm according to International Myeloma Working Group (IMWG) response criteria,[1] during or after the study treatment prior to the start of subsequent antimyeloma therapy.

Progression-free survival was defined as the duration from the date of randomization to disease progression or death, whichever came first. Disease progression was determined according to the IMWG criteria.[1] Patients who started subsequent antimyeloma therapies for multiple myeloma without disease progression were censored at the last disease assessment before the start of subsequent therapies. Patients with disease progression or death immediately preceded by two or more consecutive missed disease assessments were censored at the last disease assessment before the consecutive missed disease assessments. Patients who withdrew consent from the study before disease progression were censored at the last disease assessment. Patients who were lost to follow-up were censored at the last disease assessment before patients were lost to follow-up. Patients who had not progressed and were still alive at the cutoff date for the analysis were censored at the last disease assessment. Patients without any post-baseline disease assessment were censored at the date of randomization.

Sustained MRD-negativity rate was defined as the proportion of patients who achieved complete response or better and MRD-negative status (10^{-5}) at two examinations a minimum of one year apart (and the two examinations should be prior to disease progression, subsequent antimyeloma therapy, or both), without MRD-positive status in between.

Overall response rate was defined as the proportion of patients achieving partial response or better (i.e., partial response, very good partial response, complete response, or stringent complete response) based on computerized algorithm in accordance with the International Myeloma Working Group (IMWG) criteria,[1] during or after the study treatment but before the start of subsequent antimyeloma therapy.

Overall survival was defined as the time from the date of randomization to the date of the patient's death due to any cause. Patients who are lost to follow-up are censored at the time of lost to follow-up. Patients who died after consent withdrawal were considered as having an overall survival event. If the patient was alive at the cutoff date for the analysis or the survival status was unknown, then the patient's data was censored at the date the patient was last known to be alive.

Progression-free survival on next line of therapy (PFS2) was defined as the time from randomization to progression on the next line of treatment or death (due to any cause), whichever came first. Disease progression was based on investigator judgement. Patients who were still alive and had not yet progressed on the next line of therapy were censored on the last date of follow-up. Patients who withdrew consent or were lost to follow-up prior to any subsequent antimyeloma therapy were censored at the date of last disease assessment during the course of study. Patients without any post-baseline follow-up were censored at randomization.

Quality of life was assessed using the European Organization for Research and Treatment of Cancer Quality of Life Questionnaire–Core 30 (EORTC QLQ-C30). The EORTC QLQ-C30 includes 30 items, within a 1-week recall, resulting in 5 functional scales (physical functioning, role functioning, emotional functioning, cognitive functioning, and social functioning), 1 Global Health Status scale, 3 symptom scales (fatigue, nausea and vomiting, and pain), and 6 single items (dyspnea, insomnia, appetite loss, constipation, diarrhea, and financial difficulties).

Supplementary Fig. 1. Progression-free Survival on Next Line of Therapy (PFS2).

Shown are the results of the Kaplan−Meier estimates of PFS2 among patients in the intention-to-treat population (Panel A) and censoring death due to COVID-19 (Panel B). A sensitivity analysis was performed to adjust for the impact of COVID-19 deaths.

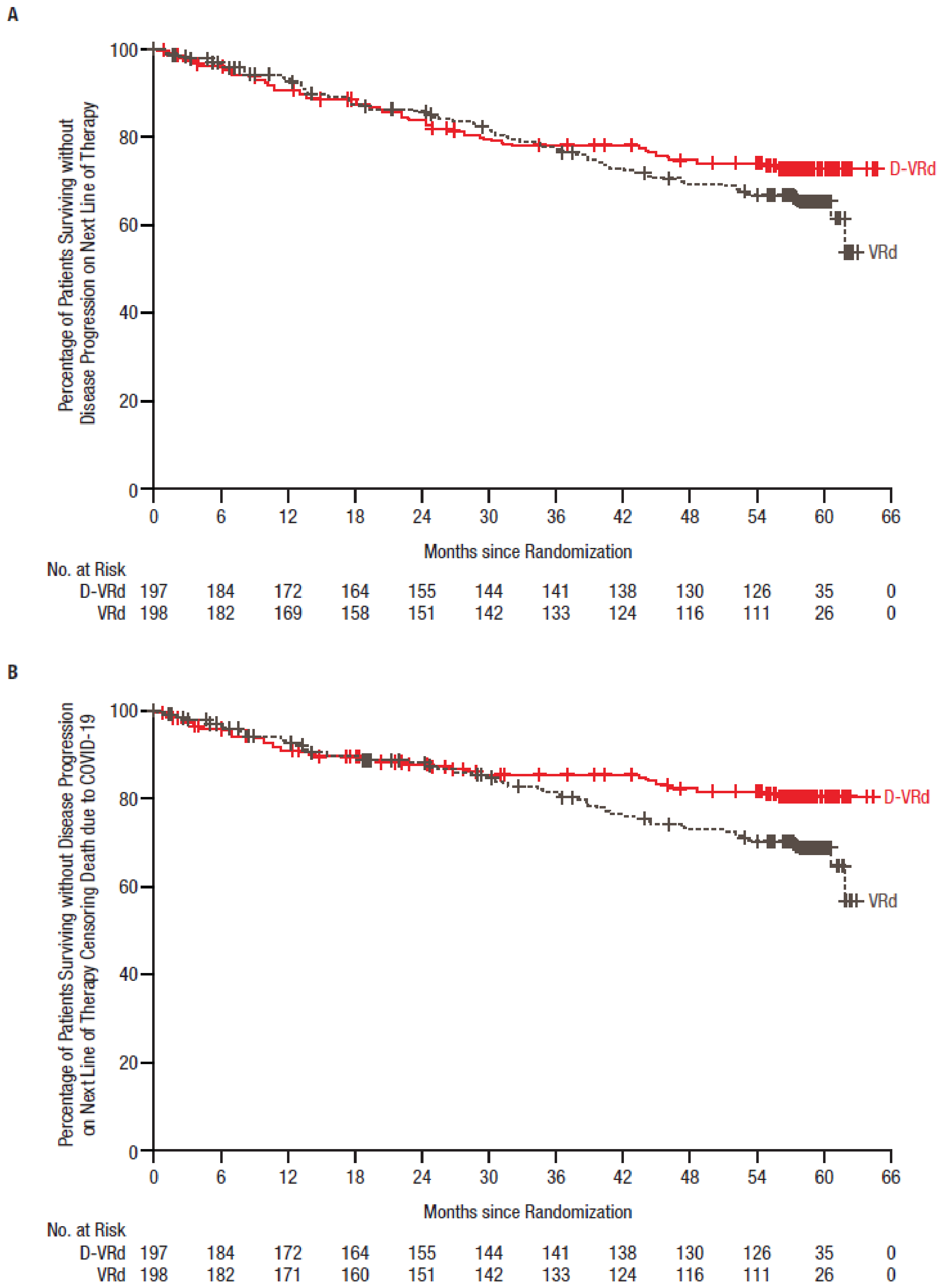

D-VRd, subcutaneous daratumumab plus bortezomib/lenalidomide/dexamethasone; VRd, bortezomib/lenalidomide/dexamethasone.

Supplementary Table 1. Study Enrollment by Region and Country in the Intention-to-Treat Population.*

Region/Country	D-VRd (n = 197)	VRd (n = 198)	Total (N = 395)
Europe Region – no. (%)	120 (60.9)	116 (58.6)	236 (59.7)
Poland	37 (18.8)	37 (18.7)	74 (18.7)
Turkey	22 (11.2)	17 (8.6)	39 (9.9)
France	18 (9.1)	14 (7.1)	32 (8.1)
Czech Republic	14 (7.1)	11 (5.6)	32 (8.1)
Spain	11 (5.6)	8 (4.0)	19 (4.8)
United Kingdom	8 (4.1)	17 (8.6)	25 (6.3)
Israel	8 (4.1)	7 (3.5)	15 (3.8)
Netherlands	2 (1.0)	4 (2.0)	6 (1.5)
Germany	0	1 (0.5)	1 (0.3)
North America – no. (%)	37 (18.8)	31 (15.7)	68 (17.2)
Canada	20 (10.2)	21 (10.6)	41 (10.4)
United States	17 (8.6)	10 (5.1)	27 (6.8)
Other – no. (%)	40 (20.3)	51 (25.8)	91 (23.0)
Brazil	31 (15.7)	38 (19.2)	69 (17.5)
Japan	9 (4.6)	13 (6.6)	22 (5.6)

D-VRd, subcutaneous daratumumab plus bortezomib/lenalidomide/dexamethasone; VRd, bortezomib/lenalidomide/dexamethasone.
*The intention-to-treat population was defined as all patients who underwent randomization.

Supplementary Table 2. Summary of Total COVID-19 Deaths and COVID-19 Serious Adverse Events By Year in the Safety Population.*

	D-VRd (n = 197)		VRd (n = 195)		Total (n = 392)	
	COVID-19 deaths	COVID-19 SAEs	COVID-19 deaths	COVID-19 SAEs	COVID-19 deaths	COVID-19 SAEs
Patients with COVID-19 deaths/serious adverse events – no. (%)	15 (7.6)	30 (15.2)†	9 (4.6)	20 (10.3)	24 (6.1)	50 (12.8)†
Proportion of COVID-19 deaths/serious adverse events by calendar year – no. (%)‡						
2020	3 (20.0)	10 (33.3)	1 (11.1)	6 (30.0)	4 (16.7)	16 (32.0)
2021	10 (66.7)	12 (40.0)	7 (77.8)	9 (45.0)	17 (70.8)	21 (42.0)
2022	2 (13.3)	7 (23.3)	1 (11.1)	4 (20.0)	3 (12.5)	11 (22.0)
2023	0	1 (3.3)	0	1 (5.0)	0	2 (4.0)
2024	0	1 (3.3)	0	0	0	1 (2.0)

D-VRd, subcutaneous daratumumab plus bortezomib/lenalidomide/dexamethasone; SAE, serious adverse event; VRd, bortezomib/lenalidomide/dexamethasone.

*The safety population included patients who received at least one dose of study treatment.

†SAE occurrence includes one patient who had a COVID-19 SAE in 2021 and 2022.

‡Percentages per year calculated with the number of patients with COVID-19 deaths/SAEs in the corresponding column as the denominator.

Supplementary Table 3. Summary of Treatment Modifications and Discontinuations in the Safety Population.*

	D-VRd (n = 197)	VRd (n = 195)
Cycle delays – no. (%)	177 (89.8)	168 (86.2)
Bortezomib		
Dose reduction	67 (34.0)	84 (43.1)
Dose delays	5 (2.5)	5 (2.6)
Dose skipped	145 (73.6)	139 (71.3)
Discontinuation	25 (12.7)	32 (16.4)
Lenalidomide		
Dose reduction	130 (66.0)	138 (70.8)
Dose skipped	149 (75.6)	135 (69.2)
Discontinuation	63 (32.0)	48 (24.6)
Dexamethasone		
Dose reduction	128 (65.0)	127 (65.1)
Dose skipped	155 (78.7)	143 (73.3)
Discontinuation	47 (23.9)	69 (35.4)
Daratumumab		
Dose delays	44 (22.3)	
Dose skipped	83 (42.1)	
Discontinuation	34 (17.3)	

D-VRd, subcutaneous daratumumab plus bortezomib/lenalidomide/dexamethasone; VRd, bortezomib/lenalidomide/dexamethasone.
*The safety population included patients who received at least one dose of study treatment.

Supplementary Table 4. Summary of Patients With Treatment Discontinuation or Dose Modification Due to Treatment-emergent Peripheral Neuropathy in the Safety Population.*

	D-VRd (n = 197)			VRd (n = 195)		
	All grades	Grade 2	Grade 3 or 4	All grades	Grade 2	Grade 3 or 4
Discontinuation of – no. (%)						
All study treatment	16 (8.1)	7 (3.6)	8 (4.1)	19 (9.7)	11 (5.6)	8 (4.1)
Bortezomib	14 (7.1)	6 (3.0)	8 (4.1)	17 (8.7)	10 (5.1)	7 (3.6)
Dose modification of – no. (%)						
Any study treatment	12 (6.1)	6 (3.0)	6 (3.0)	15 (7.7)	10 (5.1)	5 (2.6)
Bortezomib	11 (5.6)	5 (2.5)	6 (3.0)	14 (7.2)	10 (5.1)	4 (2.1)

D-VRd, subcutaneous daratumumab plus bortezomib/lenalidomide/dexamethasone; VRd, bortezomib/lenalidomide/dexamethasone.
*The safety population included patients who received at least one dose of study treatment. Peripheral neuropathy is a combined group term that includes peripheral sensory neuropathy, peripheral motor neuropathy, peripheral sensorimotor neuropathy, neuropathy peripheral, and polyneuropathy.

Supplementary Table 5. Summary of Second Primary Malignancies in the Safety Population.*

	D-VRd (n = 197)	VRd (n = 195)	Total (N = 392)
Total no. of patients with new malignancy – no. (%)	15 (7.6)	18 (9.2)	33 (8.4)
Cancer type/dictionary-derived term – no. (%)			
Cutaneous	7 (3.6)	7 (3.6)	14 (3.6)
Basal cell carcinoma	3 (1.5)	2 (1.0)	5 (1.3)
Squamous cell carcinoma	2 (1.0)	3 (1.5)	5 (1.3)
Squamous cell carcinoma of skin	2 (1.0)	1 (0.5)	3 (0.8)
Bowen's disease	1 (0.5)	1 (0.5)	2 (0.5)
Carcinoma in situ of skin	1 (0.5)	0	1 (0.3)
Kaposi's sarcoma	1 (0.5)	0	1 (0.3)
Malignant melanoma	0	1 (0.5)	1 (0.3)
Malignant melanoma in situ	0	1 (0.5)	1 (0.3)
Malignant melanoma stage II	0	1 (0.5)	1 (0.3)
Metastatic squamous cell carcinoma	0	1 (0.5)	1 (0.3)
Squamous cell carcinoma of the oral cavity	0	1 (0.5)	1 (0.3)
Hematologic	1 (0.5)	1 (0.5)	2 (0.5)
Myelodysplastic syndrome	1 (0.5)	0	1 (0.3)
Acute myeloid leukemia	0	1 (0.5)	1 (0.3)
Noncutaneous	7 (3.6)	10 (5.1)	17 (4.3)
Adenocarcinoma of colon	1 (0.5)	1 (0.5)	2 (0.5)
Transitional cell carcinoma	1 (0.5)	1 (0.5)	2 (0.5)
Bladder cancer	1 (0.5)	0	1 (0.3)
Breast cancer	1 (0.5)	0	1 (0.3)
Clear cell renal cell carcinoma	1 (0.5)	0	1 (0.3)
Intraductal proliferative breast lesion	1 (0.5)	0	1 (0.3)
Esophageal adenocarcinoma	1 (0.5)	0	1 (0.3)
Prostate cancer	0	4 (2.1)	4 (1.0)
Cholangiocarcinoma	0	1 (0.5)	1 (0.3)
Colon cancer	0	1 (0.5)	1 (0.3)
Colon neoplasm	0	1 (0.5)	1 (0.3)
Neoplasm malignant	0	1 (0.5)	1 (0.3)

D-VRd, subcutaneous daratumumab plus bortezomib/lenalidomide/dexamethasone; VRd, bortezomib/lenalidomide/dexamethasone.
*The safety population included patients who received at least one dose of study treatment.

References

1. Rajkumar SV, Harousseau JL, Durie B, et al. Consensus recommendations for the uniform reporting of clinical trials: report of the International Myeloma Workshop Consensus Panel 1. Blood 2011;117:4691-5.
