# Supplementary material for: Daratumumab plus bortezomib, lenalidomide and dexamethasone for transplant-ineligible or transplant-deferred newly diagnosed multiple myeloma: the randomized phase 3 CEPHEUS trial
Source: Nat Med. 2025 Feb 5;31(4):1195–202. doi: 10.1038/s41591-024-03485-7 (PMC12003169; doi:10.1038/s41591-024-03485-7)
Supplement: Supplementary file 2 — Reporting Summary [file 41591_2024_3485_MOESM2_ESM.pdf]

nature portfolio

Corresponding author(s): Saad Usmani

Last updated by author(s): Dec 12, 2024

Reporting Summary

Nature Portfolio wishes to improve the reproducibility of the work that we publish. This form provides structure for consistency and transparency in reporting. For further information on Nature Portfolio policies, see our Editorial Policies and the Editorial Policy Checklist.

Statistics

For all statistical analyses, confirm that the following items are present in the figure legend, table legend, main text, or Methods section.

n/a	Confirmed	
☐	☒	The exact sample size (*n*) for each experimental group/condition, given as a discrete number and unit of measurement
☐	☒	A statement on whether measurements were taken from distinct samples or whether the same sample was measured repeatedly
☐	☒	The statistical test(s) used AND whether they are one- or two-sided<br>*Only common tests should be described solely by name; describe more complex techniques in the Methods section.*
☐	☒	A description of all covariates tested
☐	☒	A description of any assumptions or corrections, such as tests of normality and adjustment for multiple comparisons
☐	☒	A full description of the statistical parameters including central tendency (e.g. means) or other basic estimates (e.g. regression coefficient) AND variation (e.g. standard deviation) or associated estimates of uncertainty (e.g. confidence intervals)
☐	☒	For null hypothesis testing, the test statistic (e.g. *F*, *t*, *r*) with confidence intervals, effect sizes, degrees of freedom and *P* value noted<br>*Give P values as exact values whenever suitable.*
☒	☐	For Bayesian analysis, information on the choice of priors and Markov chain Monte Carlo settings
☐	☒	For hierarchical and complex designs, identification of the appropriate level for tests and full reporting of outcomes
☒	☐	Estimates of effect sizes (e.g. Cohen's *d*, Pearson's *r*), indicating how they were calculated

Our web collection on statistics for biologists contains articles on many of the points above.

Software and code

Policy information about availability of computer code

Data collection	The CEPHEUS study used electronic Case Report Forms (eCRFs) to collect data from the participants (Version 9.00, 08JUN2022 MH).
Data analysis	All statistical analyses were performed using SAS Software 9.4 (TS1M6 MBCS3170).

For manuscripts utilizing custom algorithms or software that are central to the research but not yet described in published literature, software must be made available to editors and reviewers. We strongly encourage code deposition in a community repository (e.g. GitHub). See the Nature Portfolio guidelines for submitting code & software for further information.

Data

Policy information about availability of data

All manuscripts must include a data availability statement. This statement should provide the following information, where applicable:

- Accession codes, unique identifiers, or web links for publicly available datasets
- A description of any restrictions on data availability
- For clinical datasets or third party data, please ensure that the statement adheres to our policy

The data sharing policy of Janssen Pharmaceutical Companies of Johnson & Johnson is available at https://www.janssen.com/clinical-trials/transparency. As noted on this site, requests for access to the study data can be submitted through Yale Open Data Access (YODA) Project site at http://yoda.yale.edu. The trial protocol and statistical analysis plan can be found in the Supplementary Appendix.

Research involving human participants, their data, or biological material

Policy information about studies with human participants or human data. See also policy information about sex, gender (identity/presentation), and sexual orientation and race, ethnicity and racism.

Reporting on sex and gender	Sex was collected and reported in the trial; sex was reported by the patient. See Table 1 (Demographics).
Reporting on race, ethnicity, or other socially relevant groupings	Race was reported by the patient. See Table 1 (Demographics).
Population characteristics	Eligible patients had transplant-ineligible or transplant-deferred NDMM. See Table 1 (Demographics).
Recruitment	This randomized, open-label, multicenter phase 3 study enrolled patients between December 11, 2018 and October 7, 2019 at 92 sites in 13 countries (Supplementary Appendix). Eligible patients had transplant-ineligible or transplant-deferred NDMM, an ECOG performance status score of 0-2, and a frailty index <2 (see Methods section for full eligibility criteria). Patients were randomly (1:1) assigned to D-VRd or VRd by randomly permuted blocks using an interactive web-response system. Randomization was stratified by ISS disease stage (I, II, or III) and age/transplant eligibility (<70 years ineligible, <70 years and transplant deferred, or ≥70 years). There was no selection of patients.
Ethics oversight	An independent ethics committee or institutional review board approved the protocol at each site. The study was conducted in accordance with the International Conference on Harmonisation Good Clinical Practice guidelines, the principles originating from the Declaration of Helsinki, and study site-specific regulations. All patients provided written informed consent.

Note that full information on the approval of the study protocol must also be provided in the manuscript.

Field-specific reporting

Please select the one below that is the best fit for your research. If you are not sure, read the appropriate sections before making your selection.

☒ Life sciences ☐ Behavioural & social sciences ☐ Ecological, evolutionary & environmental sciences

For a reference copy of the document with all sections, see nature.com/documents/nr-reporting-summary-flat.pdf

Life sciences study design

All studies must disclose on these points even when the disclosure is negative.

Sample size	We estimated that a sample size of 360 patients (180 in each arm) was needed to achieve 80% power to detect a 15% treatment difference in overall MRD-negativity rate at a two-sided alpha of 0.05. This sample size would also provide 80% power to detect a 37% reduction in the risk of disease progression or death with a log-rank test at a two-sided alpha of 0.05.
Data exclusions	No data exclusions were performed.
Replication	Not applicable (clinical trial).
Randomization	Patients were randomly (1:1) assigned to D-VRd or VRd by randomly permuted blocks using an interactive web-response system. Randomization was stratified by ISS disease stage (I, II, or III) and age/transplant eligibility (<70 years ineligible, <70 years and transplant deferred, or ≥70 years).
Blinding	This was an open-label study; blinding procedures are not applicable.

Reporting for specific materials, systems and methods

We require information from authors about some types of materials, experimental systems and methods used in many studies. Here, indicate whether each material, system or method listed is relevant to your study. If you are not sure if a list item applies to your research, read the appropriate section before selecting a response.

Materials & experimental systems

n/a	Involved in the study
☒	☐ Antibodies
☒	☐ Eukaryotic cell lines
☒	☐ Palaeontology and archaeology
☒	☐ Animals and other organisms
☐	☒ Clinical data
☒	☐ Dual use research of concern
☒	☐ Plants

Methods

n/a	Involved in the study
☒	☐ ChIP-seq
☒	☐ Flow cytometry
☒	☐ MRI-based neuroimaging

Clinical data

Policy information about clinical studies

All manuscripts should comply with the ICMJE guidelines for publication of clinical research and a completed CONSORT checklist must be included with all submissions.

Clinical trial registration

ClinicalTrials.gov identifier: NCT03652064.

Study protocol

Redacted protocol provided

Data collection

The study enrolled patients between December 11, 2018 and October 7, 2019 at 92 sites in 13 countries. This study was sponsored by Janssen Research & Development, LLC. The sponsor and investigators designed the trial and compiled, maintained, and analyzed the data collected by the investigators throughout the study until the clinical cutoff date (May 7, 2024). The full listing of participating centers and primary investigators is available in the Supplementary Appendix. The CEPHEUS study used electronic Case Report Forms (eCRFs) to collect data from the participants (Version 9.00, 08JUN2022 MH).

Outcomes

The primary endpoint was overall MRD-negativity rate, defined as the proportion of patients who achieved ≥CR and had MRD-negative status (at or below a sensitivity threshold of 10–5) after randomization but prior to progression, subsequent antimyeloma therapy, or both. Major secondary endpoints were ≥CR rate, PFS, and sustained MRD-negativity rate (≥12 months). ≥CR rate was defined as the proportion of patients achieving CR or stringent CR during or after the study treatment prior to the start of subsequent antimyeloma therapy. PFS was defined as the duration from the date of randomization to disease progression or death, whichever came first. Sustained MRD-negativity rate was defined as the proportion of patients who achieved ≥CR and MRD-negative status (10–5) at two examinations a minimum of one year apart (and the two examinations should be prior to disease progression, subsequent antimyeloma therapy, or both), without MRD-positive status in between.

MRD was evaluated via next-generation sequencing using the clonoSEQ assay (v.2.0; Adaptive Biotechnologies, Seattle, WA) using bone marrow aspirate samples obtained at baseline, at the time of suspected complete response, and at 12, 18, 24, 30, and 36 months after the first dose and annually thereafter in patients who achieved confirmed complete response. Tumor response and disease progression were assessed using a validated computer algorithm in accordance with IMWG response criteria 2011. Disease assessments were performed at a central laboratory. TEAEs were graded according to the NCI-CTCAE version 5.0. TEAEs were reported until 30 days following the last dose of any component of the treatment regimen.

Plants

Seed stocks

Report on the source of all seed stocks or other plant material used. If applicable, state the seed stock centre and catalogue number. If plant specimens were collected from the field, describe the collection location, date and sampling procedures.

Novel plant genotypes

Describe the methods by which all novel plant genotypes were produced. This includes those generated by transgenic approaches, gene editing, chemical/radiation-based mutagenesis and hybridization. For transgenic lines, describe the transformation method, the number of independent lines analyzed and the generation upon which experiments were performed. For gene-edited lines, describe the editor used, the endogenous sequence targeted for editing, the targeting guide RNA sequence (if applicable) and how the editor was applied.

Authentication

Describe any authentication procedures for each seed stock used or novel genotype generated. Describe any experiments used to assess the effect of a mutation and, where applicable, how potential secondary effects (e.g. second site T-DNA insertions, mosiacism, off-target gene editing) were examined.
